# Engineering strategies of sequential drug delivery systems for combination tumor immunotherapy

**DOI:** 10.1016/j.apsb.2025.05.039

**Published:** 2025-06-06

**Authors:** Zhenyu Xu, Siyan Liu, Yanan Li, Yanping Wu, Jiasheng Tu, Qian Chen, Chunmeng Sun

**Affiliations:** a State Key Laboratory of Natural Medicines, China Pharmaceutical University, Nanjing 211198, China; b Department of Pharmaceutics, School of Pharmacy, China Pharmaceutical University, Nanjing 211198, China; c Jiangsu Key Laboratory of TCM Evaluation and Translational Research, and Research Center for Traceability and Standardization of TCMs, School of Traditional Chinese Pharmacy, China Pharmaceutical University, Nanjing 211198, China; d School of Food Science and Pharmaceutical Engineering, Nanjing Normal University, Nanjing 210023, China; e School of Basic Medicine and Clinical Pharmacy, China Pharmaceutical University, Nanjing 211198, China; f Jiangsu Provincial Key Laboratory of Drug Metabolism and Pharmacokinetics, China Pharmaceutical University, Nanjing 210009, China; g Wuxi Research Center for Innovative Medicines and Life Health, Wuxi 214122 China

**Keywords:** Sequential release, Spatiotemporally-tuned delivery, Combination therapy, Biomaterials, Tumor immunotherapy, Cancer-immunity cycle, Local drug delivery, Systemic drug delivery

## Abstract

Over the past few decades, tumor immunotherapy has revolutionized the landscape of cancer clinical treatment. There is a flourishing development of combination strategies to improve the anti-tumor efficacy of mono-immunotherapy. However, instead of a straightforward combination of multiple therapeutics, it is more preferable to pursue a synergistic effect by designing rational combinations as well as administration strategies, which are based on a comprehensive understanding of the physiological and pathological features. In this case, the timing and spatial distribution of the combination drugs become essential factors in achieving improved therapeutic outcomes. Therefore, the concept of Sequential Drug Delivery System (SDDS) is proposed to define the spatiotemporally programmed drug delivery/release through triggers of internal conditions and/or external interventions, thus complying with the dynamic disease evolution and the human immunity. This review summarizes the recent advancements in biomaterial-based SDDSs used for spatiotemporally-tuned combination tumor immunotherapy. Furthermore, the rationales behind various engineering strategies are discussed. Finally, an overview of potential synergistic mechanisms as well as their prospects for combination immunotherapy is presented.

## Graphical abstract

This review discusses the rationale of sequential drug release for spatiotemporally-tuned combination immunotherapy, reviewing different strategies based on local and systemic drug release systems.

**Figure:**
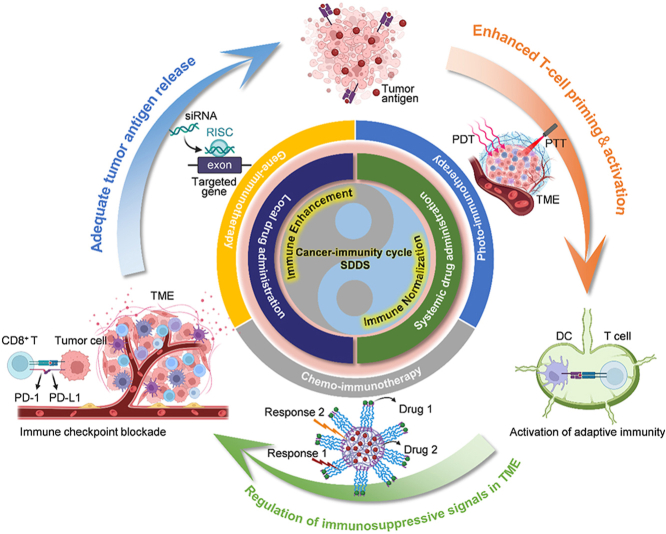

## 1 Introduction

Cancer has emerged as a significant threat to human health. In 2020, there were 19.3 million new cancer cases and 10 million cancer deaths worldwide, as reported by the International Agency for Research on Cancer (IARC)^1^. To address this severe global public health issue, several therapies have been developed, such as surgical resection^2^, ^3^, ^4^, chemotherapy^5^, ^6^, ^7^, radiotherapy^8^, ^9^, ^10^, ^11^, ^12^, and immunotherapy^13^, ^14^, ^15^, ^16^, ^17^, While the first three conventional treatments have been referred to as the “troika” of clinical cancer treatments, they can also cause significant harm to the organism. Immunotherapy provides a less toxic, longer-lasting, and more potent approach by activating and enhancing the body’s immune system to combat cancer. However, this emerging treatment has certain limitations that demand immediate attention, including low response rates^18^, ^19^, ^20^, ^21^, and the occurrence of immune-related adverse events (irAEs)^22^, ^23^, ^24^, ^25^, Moreover, many patients eventually develop secondary resistance to monotherapy in immunotherapy^26^, ^27^, ^28^, ^29^. Various strategies for combination immunotherapy have been proposed to mitigate these unfavorable circumstances^30^, ^31^, ^32^, ^33^.

**Figure 1:**
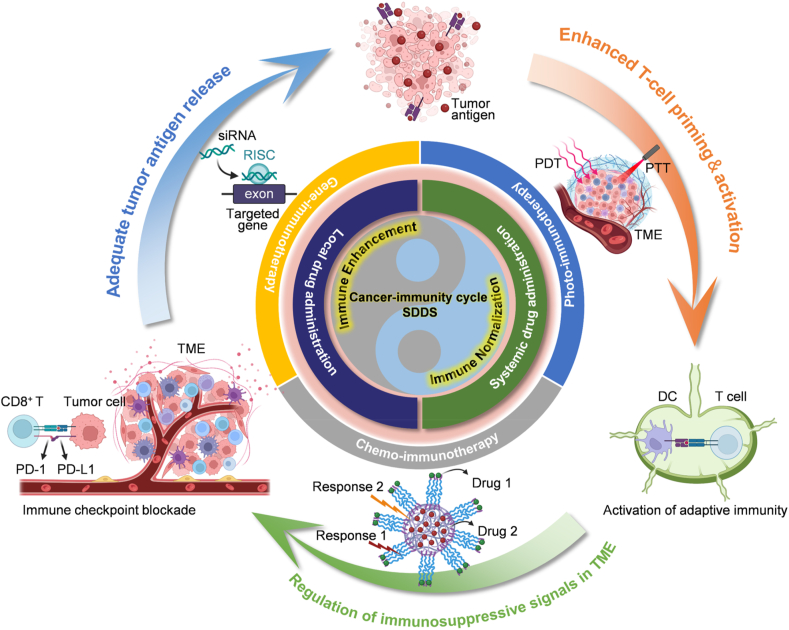
Schematic illustration of SDDS-based strategies for improving tumor immunotherapy. SDDSs have multiple delivery routes. In local drug delivery, SDDSs utilized the natural advantages of the administration mode in the spatial dimension to fulfill the principle of “enhanced efficacy and reduced toxicity”. For systemic administration, SDDSs delivered one or more cargoes to different target sites in a predetermined sequence, and this spatiotemporally-tuned strategy further combined different treatments to achieve anti-tumor combination immunotherapy. Regardless of the delivery approach, SDDSs enhanced various steps of the cancer–immunity cycle, which in turn directly activated the host immune system (immune enhancement) and/or remodeled immunosuppressive TME (immune normalization), and ultimately amplified the anti-tumor immune response.

Till present, the US Food and Drug Administration (FDA) has approved a number of combination therapies for treating different types of cancer^34^, ^35^, ^36^, ^37^. Moreover, there are still many ongoing clinical trials by combining immunotherapy with chemotherapy, radiotherapy, and targeted therapy. And more and more evidences indicate that such combination therapy is able to overcome the limitations of monotherapy and potentiate the anti-tumor effect. Combination therapy utilizes various therapeutic agents^21^^,^^38^, ^39^, ^40^, ^41^, ^42^. However, different drugs can target diverse cellular components (*e.g.*, tumor cells or immune cells in the tumor microenvironment (TME)), subcellular compartments (*e.g.*, cell membrane or cytoplasm), or both. Moreover, in addition to potential synergistic effects, the sequential release of medications may lead to antagonistic effects. To overcome these challenges, the concept of Sequential Drug Delivery System (SDDS) has been proposed. It aims to align with the dynamic processes of tumorigenesis, development, recurrence, and metastasis by artificially designing precise mechanisms or methods for the spatiotemporally controlled release of multiple medications. The fundamental goal of tumor immunotherapy is to enhance the cancer–immunity cycle, and SDDSs can interact with this cycle *in vivo*, maximizing the anti-tumor effect while minimizing off-target toxicities of the drug (Fig. 1). In recent years, several SDDSs have been developed using diverse biomaterials, leveraging their unique advantages, such as excellent biocompatibility and bioactivity. Hence, comprehending the rational design of biomaterial-based SDDSs for immunotherapy is essential for achieving precise regulation of anti-tumor immunity.

In this review, the relationship between biomaterial-based SDDSs and combination tumor immunotherapy was first analyzed, followed by a summary and discussion of biomaterial-based carriers in tumors for systemic administration and local administration in recent years, including the impact of SDDSs on the endogenous anti-tumor immunity. It should be noted that although some studies have used combination therapy, the significance of the underlying sequential principles has not been emphasized by researchers. This aspect is elucidated here. Lastly, the concluding section summarized and envisioned the future of this emerging field, and offered new perspectives to steer the development and application of SDDSs. These insights have the potential to enhance therapeutic efficiency, suppress tumor progression, recurrence, and metastasis, while also presenting opportunities and challenges for future research.

## 2 Association between combination tumor immunotherapy and sequential drug delivery systems

Tumor immunotherapy has made striking progress and altered the trajectory of cancer therapy^43^^,^^44^. Various strategies for tumor immunotherapy that engage both innate and adaptive immunity have been investigated^45^, including chimeric antigen receptor T cell therapies^46^, ^47^, ^48^, ^49^, immune checkpoint inhibitors (ICIs)^50^, ^51^, ^52^, ^53^, immunostimulatory cytokines^54^, ^55^, ^56^, ^57^, and pattern recognition receptor (PRR) agonists^58^, ^59^, ^60^, ^61^, ^62^. Tumor combination immunotherapy combines immunotherapy with other treatments like chemotherapy, radiotherapy, photodynamic therapy, photothermal therapy, and gene therapy, each using different mechanisms. Combination immunotherapy effectively restarts and maintains the cancer–immunity cycle^19^^,^^63^^,^^64^, strengthening the patient’s immune response against the tumor. Combination immunotherapy offers improved efficacy, reduced drug resistance, broader applications, and enhanced immune memory compared to monotherapy. Sequential drug delivery systems release multiple drugs in a controlled sequence from the same platform, providing precise control over the spatial and temporal level of drug release to better match dynamic disease processes. However, the limited understanding of tumor immune-combination therapy mechanisms and the design of sequential drug delivery systems has led to few SDDS-based strategies in clinical cancer treatment. Therefore, there is an urgent need to clarify how SDDS design principles can be applied to tumor combination immunotherapy.

### 2.1 Therapeutic targets of SDDS in the cancer–immunity cycle

**Figure 2:**
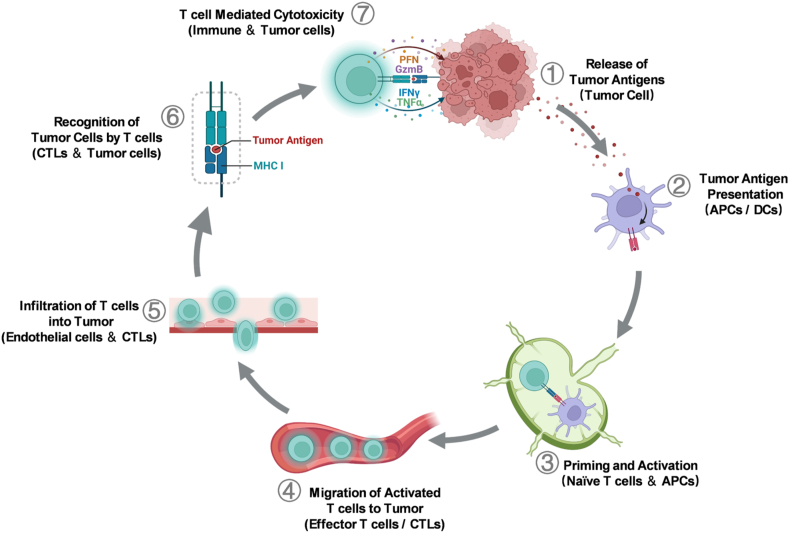
The cancer–immunity cycle. The cancer–immunity cycle can amplify the T cell response by accumulating immune stimulators. Another feature was the creation of an immunomodulatory feedback mechanism through inhibitory factors, which can limit immunity. This cycle can be divided into seven main steps, starting with the release of antigens and ending with the killing of tumor cells. The diagram above depicts each step, the main cell types involved and the location of the activity. Abbreviations: CTLs, cytotoxic T lymphocytes.

The cancer–immunity cycle, introduced by Chen and Mellan^63^ in 2013, elucidates the mechanisms by which the immune system destroys cancer cells. Tumor immunity is a dynamic cyclic process of ongoing self-derivation that accumulates immunostimulatory molecules to launch a clinically effective T cell-mediated immune response to eliminate tumors. The cancer–immunity cycle is comprised of seven distinct phases: (1) the release of tumor antigens by apoptotic or necrotic cancer cells, (2) the uptake and presentation of tumor antigens by antigen presenting cells (APCs), (3) priming and activation of naive T cells in lymphoid tissues by APCs, (4) migration of activated T cells to tumor tissues *via* blood, (5) infiltration of T cells into tumors, (6) specific recognition by effector T cells presenting homologous antigen to tumor cells, (7) targeted destruction of cancer cells by effector T cells, release of tumor antigens, and cycle resumption (Fig. 2). At each step, there are positive and negative regulatory factors that ensure the maintenance of immune system activity within normal boundaries^63^.

Cancer cells have developed defenses to counteract each step of this cycle. Once the integrity of this cycle is disrupted, a deficiency in anti-tumor immunity occurs. The immune escape of tumor cells in turn leads to the genesis and progression of cancer^65^^,^^66^. These mechanisms in detail are divided into three main categories, the first of which is defective tumor antigen release. Ineffective antigen release will prevent effective antigen presentation, which will hinder T cells from effective recognition of the tumor. For example, neoantigens and antigenic structures can affect APCs uptake^67^^,^^68^, and acquired mutations in tumor cell *β*2 microglobulin or loss of human leukocyte antigen (HLA) alleles can result in restricted antigen presentation and T cell recognition^69^. Impaired T cell priming and activation in the lymph nodes falls into the second category. Since most tumor antigens are derived from autoantigens, co-stimulatory factors and pro-inflammatory cytokines are necessary for the antigen recognition process of T cells. Without these factors, the priming and activation of regulatory T cells may occur instead of effector T cells^70^, ^71^, ^72^. The third category encompasses immunosuppressive signals in the TME. Tumors are complex structures composed of multiple cell types that can create an evolving immunosuppressive TME. These solid structures can secrete various immunosuppressive factors (*e.g.*, interleukin-6 (IL-6), IL-10, and transforming growth factor-*β* (TGF-*β*)) to suppress specific effector T cell responses or induce T cell depletion^73^, ^74^, ^75^, ^76^. Additionally, some of these cytokines can function as immunomodulatory molecules, increasing the abundance of immunosuppressive cells in the TME, such as myeloid-derived suppressor cells (MDSCs) and M2-like macrophages, thereby promoting immune escape^77^, ^78^, ^79^, ^80^, ^81^. Moreover, tumor cells or tumor-infiltrating lymphocytes can express suppressive immune checkpoints, including PD-1/PD-L1, TIGIT, LAG-3, and TIM-3 to attenuate anti-tumor immunity^82^, ^83^, ^84^, ^85^.

To overcome the tumor immune escape mechanism described above, researchers have developed numerous strategies that target various cell types within the TME^86^, ^87^, ^88^. In the case of cancer cells, the induction of immunogenic cell death (ICD) will enhance tumor immunogenicity and elicit an anti-tumor immune response by triggering adaptive immune cells^89^, ^90^, ^91^, ^92^. Similarly, the replenishment and reactivation of APCs in TME can stimulate a systemic anti-tumor T cell response^93^, ^94^, ^95^, ^96^. These two strategies are considered as “immune enhancement” since they directly activate the immune system^97^. Moreover, approaches that target the inhibition of tumor-associated immunosuppressive cells' homing, expansion, and effector function can also improve the anti-tumor response. This strategy of weakening tumor immunosuppression and remodeling TME is regarded as “immune normalization”^97^, ^98^, ^99^. The former approach mainly targets the first and second categories of immune escape mechanisms, while the latter focuses on the third category. However, mono-immunotherapy remains ineffective, which is largely attributed to adaptive immune resistance (AIR)^29^. Therefore, future combination therapy regimens should be designed based on the targets of immune normalization and immune enhancement.

The cancer–immunity cycle is a dynamic process that occurs in the spatiotemporal dimension. Therefore, it is challenging to fulfill all the requirements of the *in vivo* immune effector process using a drug delivery system with a single release behavior. Traditional combination therapy with cocktail drugs usually faces significant limitations due to coordination issues as they do not consider variations in the pharmacokinetics and active sites of individual drugs. Moreover, precise delivery and on-demand release of immunotherapeutic agents at the intended site can effectively decrease the incidence of irAEs. Therefore, it is crucial to develop a sequential delivery carrier that can fulfill the temporal and spatial requirements of each drug to ensure the integrity and durability of the cancer–immunity cycle. As an integrated platform for multiple drugs or therapeutic strategies, SDDSs can sequentially activate disrupted steps in the cancer–immune cycle. For instance, the initial release of drugs can achieve immune enhancement by triggering ICD, restoring defective tumor antigen release (step 1), tumor antigen presentation (step 2) and impaired T-cell priming and activation (step 3). Subsequently released drugs reverses the immunosuppressive signals in TME (step 6), *i.e.*, immune normalization, further amplifying the anti-cancer response synergistically. In summary, different steps of the cancer–immunity cycle can be targeted by SDDSs to produce a cascade of synergistic antitumor effects.

### 2.2 Sequential drug delivery system and its design strategies

Sequential drug release (SDR) involves releasing drugs into the body in a predetermined time sequence and rate. This technique is commonly used in situations where different drugs need to be released at different stages or the same drug at different rates. Generally, SDR-based drug delivery platforms are referred to as sequential drug delivery systems (SDDSs). SDDS differs from sequential drug therapy (SDT) as they are distinct in concept and application. SDT is a strategy that typically involves the sequential use of different drugs to achieve a treatment goals. In clinical oncology immuno-SDT, a physician may tailor a personalized treatment regimen based on disease progression, drug response, and sequencing of the patient’s genes, and subsequently administer the drugs involved in the regimen in chronological order^100^, ^101^, ^102^, ^103^. Common SDT strategies include using chemotherapeutic agents followed by immune checkpoint inhibitors, or combining various immunotherapeutic agents. Therefore, tumor immuno-SDT is a form of combination tumor immunotherapy.

SDDS, a platform that integrates multiple drugs, provides significant clinical benefits compared to the traditional method of administering drugs sequentially through different delivery routes: (1) Precise timing control: SDDS precisely controls drug release timing, ensuring optimal therapeutic concentrations at the ideal moment, which enhances therapeutic efficacy and minimizes side effects. (2) Simplification of treatment regimens: By managing multiple drugs in one delivery system, SDDS simplifies treatment, crucial for chronic conditions needing long-term medication. (3) Enhancing patient compliance: SDDS improves quality of life by making drug administration less frequent and simpler, particularly for those who struggle with self-administration, like the elderly or cognitively impaired. (4) Reducing drug interaction risks: SDDS minimizes risks by precisely controlling drug release sequences and rates, optimizing efficacy and reducing adverse reactions. (5) Targeted delivery: SDDS can target specific tissues or cells, allowing direct action at the lesion site instead of distributing it throughout the body, which could improve therapeutic precision and reduce effects on healthy tissues. (6) Improved bioavailability: Integrating drugs into one delivery system enhances bioavailability, particularly for drugs that are unstable or degraded by traditional methods.

The administration modes of SDDS are mainly categorized into systemic administration and local administration. Systemic drug administration exerts its effects through systemic circulation, and its advantages include: (1) broad action: it can affect multiple sites throughout the body, including hard-to-reach metastatic tumors; (2) sustained therapy: it ensures the continuity and stability of the therapeutic effect by maintaining a constant blood concentration of the drug; and (3) easy to administer: it is usually administered orally or intravenously, which makes it easy to be clinically operated and administered. However, systemic administration may produce adverse effects on non-target organs, such as autoimmune diseases that may be triggered by an overactive immune system. In addition, uneven distribution of drug efficacy is a limitation, especially in the tumor microenvironment, where the drug may not be able to effectively accumulate at the lesion site. Systemic drug administration is mainly indicated for advanced or widely metastatic tumors and for situations that are difficult to control by surgery or local therapy. Local drug administration, on the other hand, involves direct application of the drug to the lesion, and commonly used methods include implanted devices or local injection. Advantages include: (1) precise targeting: direct action on the tumor site improves local drug concentration and enhances therapeutic effect; (2) lower side effects: local action reduces systemic exposure, thus lowering toxicity; and (3) dosage optimization: higher dosages can be used without increasing the risk of systemic side effects. However, local administration is limited to accessible tumors, with reduced effectiveness for deep or metastatic tumors. Moreover, it requires technical expertise, specialized equipment, and is often costly. Despite these challenges, SDDS aims to address these limitations with strategic approaches detailed in the following sections.

Design strategies for SDDSs vary significantly between systemic and local delivery due to differences in administration routes. When administered systemically, SDDS must navigate multiple biological barriers to reach the TME. This continuum includes blood circulation, tumor accumulation and penetration, cellular internalization, and eventual cargo release. The first three stages are well-documented in literature and this paper focuses on the sequential release of the cargo^104^. Generally, biomaterial-based SDDSs release cargo through triggers from both endogenous and exogenous cues. Endogenous triggers are often physiological characteristics within the TME^105^, ^106^, ^107^, such as extracellular acidic pH can lead to protonation or breakage of certain acid-sensitive chemical bonds (*e.g.*, hydrazone bonds, ester bond, schiff base bond, carboxyl group, amines); and higher intracellular redox potential can lead breakage of the redox-responsive bonds (*e.g.*, diselenide bonds, disulfide bonds, thioketal bonds, thioketone bonds); biological enzymes can also facilitate cargo release (*e.g.*, matrix metalloproteinase, hyaluronidase, cathepsin). Typically, the endogenous response is usually accompanied by the release of the cargo as well as physical changes in the carrier, such as charge transitions, shape transitions, and size transitions. Following these physical changes, another cargo is gradually released over time. Exogenous triggers for SDDSs include light, magnetic, and acoustic energy, which facilitate cargo release by responding to these stimuli. For instance, light can break photosensitive bonds; magnetism can remotely induce structural changes in magnetic carriers^108^, facilitating drug release; and ultrasound can manipulate ultrasound-responsive biomaterials such as microbubbles and bubble liposomes.

Most local administration SDDSs utilize drug-containing scaffolds. Design strategies include controlling cargo-backbone affinity *via* material chemistry (electrostatic interactions, hydrophobic–hydrophilic interactions, or tailoring the cargo’s affinity for a certain material); the solubility and degradation of the backbone (controlling the length of cross-linking agents, degradation, and density); controlling the diffusion of the cargo from the material rate (changing the scaffold’s mesh size, cargo’s molecular weight and concentration). Mesh size is adjusted by modifying the polymer’s molecular weight and concentration, cross-linking type and density, and the use of a porogenic agent. These strategies allow for precise control of drug release rates from the scaffold to achieve SDR.

In conclusion, constructing a sequential release carrier capable of overcoming the biological barrier of cancer at the organ, suborgan, or even subcellular level presents significant challenges. However, various preclinical studies have demonstrated success in sustaining the cancer–immunity cycle, as well as suppressing tumor growth and metastasis, by employing diverse administration approaches and carrier designs. These approaches will be further discussed in the subsequent section.

## 3 Application of biomaterial-based SDDSs for systemic drug delivery

Systemic administration is a straightforward, practical, cost-effective approach that does not necessitate specialized equipment. Moreover, systemic administration allows the drug to reach various tissues and organs throughout the body, which is vital for treating systemic oncological diseases or advanced tumors with distant metastases. Conventional drug delivery systems face challenges in achieving the desired outcomes due to variations in the physicochemical properties, pharmacokinetics, and tissue distribution of different drugs. Nanodrug delivery technologies enhance drug solubility, modulate drug distribution *in vivo*, and improve drug targeting. Therefore, they have addressed the aforementioned challenges and enabled further exploration of immunotherapy combinations. Developing SDDSs that exhibit stability in blood circulation and can traverse various biological barriers within tumors for programmed release at specific locations and time points presents a challenge due to the predominant use of intravenous injection in systemic administration. Upon arrival at the TME, intracellular processes are equally critical for achieving the desired efficacy, as these processes determine the different fates of the drug. Tumors display distinct physiological characteristics within the tumor cells, such as acidic pH, higher redox potential, increased hypoxia, enzyme overexpression and increased metabolic activity^109^. These properties can largely influence the intracellular processes of drugs and are relevant to the design of SDDSs^110^. Researchers have proposed several effective stimulus-responsive techniques by utilizing various types of smart materials (with variable physical and chemical properties, surface modification, and responsiveness) and employing complex structural designs. These techniques encompass pH response^111^, ^112^, ^113^, ^114^, light response^115^^,^^116^, bioenzyme response^117^, ^118^, ^119^, hypoxia-responsive^120^^,^^121^, reactive oxygen species (ROS) response^122^, ^123^, ^124^, and glutathione (GSH) response^125^, ^126^, ^127^. These responses can induce changes in carrier properties, such as surface charge inversion, size reduction, alterations in receptor binding, and hydrophilic-hydrophobic transitions, facilitating drug-specific distribution and enhancing the efficacy of combination immunotherapy in tumor settings. This section provides a summary and discussion of various systemic sequential drug delivery strategies (Table 1^112^^,^^113^^,^^115^^,^^116^^,^^118^^,^^119^^,^^122^^,^^123^^,^^128^, ^129^, ^130^, ^131^, ^132^, ^133^, ^134^, ^135^, ^136^).

**Table 1:** Recent advances in systemic administration of SDDSs in tumor immunotherapy.

Combination therapy	Therapeutics	Response and/or drug release site	Type and effect of response	Targets of cancer–immunity cycle	Ref.
Chemo-immunotherapy	DOX + R848	TME + endo-lysosomal compartment	pH response-mediated hydrophobic/hydrophilic transformation and drug release	Step 1, 2 and 3	128
Chemo-immunotherapy	aPD-L1 + PTX	TME + cytoplasm	MMP and pH response-mediated drug release	Step 1 and 6	113
Chemo-immunotherapy	aPD-1 + PTX	TME + endo-lysosomal compartment	MMP response-mediated charge reversal + aPD-1 release and pH response-mediated PTX release	Step 1 and 6	129
Chemo-immunotherapy	DOX + CpG	Cell membrane + cytoplasm	apoA-1/SR-B1-mediated dissociation of HDL and AS1441 aptamer-mediated entry into the nucleus	Step 1, 2 and 3	130
Chemo-immunotherapy	HY19991 + THZ + PTX	TME + endo-lysosomal compartment	MMP response-mediated release of outer HY + THZ and pH response-mediated release of PTX from inner micelles	Step 1, 2, 3 and 6	131
Chemo-immunotherapy	BLZ945 + Pt (Ⅳ)	Tumor perivascular + cytoplasm	Laser triggered BLZ release and carrier shrinkage	Step 1 and 6	115
Chemo-immunotherapy	RGX-104 + PTX	Tumor perivascular + endo-lysosomal compartment	Dual response of pH mediated shrinkage and drug release	Step 1 and 2	112
Chemo-immunotherapy	SB431542 + DOX + Fc	TME + cytoplasm	HSPE response-mediated drug release and charge inversion	Step 1, 2, 3 and 6	118
Photodynamic-immunotherapy	dPPA + PXTK + PheoA	TME + cytoplasm	HAase response-mediated shrinkage + dPPA release and PDT-derived ROS response-mediated drug release	Step 1, 2, 3 and 6	123
Photodynamic-immunotherapy	^D^PPA-1 + MB	TME + cytoplasm	MMP response-mediated charge reversal and ^D^PPA-1 release	Step 1, 2, 3 and 6	119
Photodynamic-immunotherapy	Ce6 + NLG919	TME + cytoplasm	HAase response increased accumulation and PDT-derived ROS response mediated structural inversion and IDOi release	Step 1, 2, 3 and 6	132
Photodynamic-immunotherapy	aPD-L1 + ZnPc	TME + cytoplasm	Dual response of pH and MMP mediated charge inversion and aPD-L1 release	Step 1, 2, 3 and 6	122
Photothermal-immunotherapy	siPD-L1 + bismuthene	TME + endo-lysosomal compartment	pH response-mediated shrinkage with charge inversion + NIR triggered endo-lysosomal escape to release siRNA	Step 1, 2, 3 and 6	133
Photothermal-immunotherapy	Epacadostat + ICG	TME + cytoplasm	MMP response-mediated shrinkage + NIR triggered cell death and IDOi release	Step 1, 2, 3 and 6	116
Gene-immunotherapy	A7R + siPD-L1 + Ce6	TME + cytoplasm	MMP response-mediated ATR release and laser-triggered drug release and PDT	Step 1, 2, 3, 5 and 6	134
Gene-immunotherapy	aPD-L1 + siCD155	Cell membrane + endo-lysosomal compartment	Receptor-mediated carrier internalization and pH response-mediated siRNA release	Step 6 and 7	38
Gene-immunotherapy	PTX + siRedd1	TME and M2-like macrophages	pH response-mediated PTX release and MR-mediated targeting of TAMs	Step 1, 2, 3 and 6	136
Gene-immunotherapy	siPD-L1 + bismuthene	TME + endo-lysosomal compartment	pH response-mediated shrinkage with charge inversion + NIR triggered endo-lysosomal escape to release siRNA	Step 1, 2, 3 and 6	133

DOX, doxorubicin; PTX, paclitaxel; TME, tumor microenvironment; MMP, matrix metalloproteinase; aPD-1, anti-programmed cell death-1; aPD-L1, anti-programmed cell death ligand-1; HDL, high density lipoprotein; CpG, oligodeoxynucleotide; apoA-1, apolipoprotein A-I; SR-B1, scavenger receptor class B type I; THZ, thioridazine; Fc, ferrocene; HSPE, heparinase; PXTK, cinnamaldehyde and thioacetal-based PTX dimer precursor; MB, methylene blue; PDT, photodynamic therapy; IDOi, indoleamine-2,3-dioxygenase inhibitor; NIR, near infrared; TAMs, tumor-associated macrophages; Redd1, Response1; siRNA, small interfering RNA.

### 3.1 Combination strategy of chemo-immunotherapy with biomaterial-based SDDSs

Chemotherapy is a highly efficient treatment for cancer. However, the high heterogeneity of tumor tissues makes them prone to developing drug-resistant mutations, which can diminish the effectiveness of chemotherapy^137^. In recent years, significant advancements in developing treatment approaches for tumor immunotherapy have been achieved. However, the low response rate, attributed to the diverse mechanisms of immune defense employed by cancer cells, remains a major challenge^138^. Some studies have demonstrated that chemotherapeutic agents can counteract the immunosuppression of TME, leading to an enhancement of the therapeutic effects of immunotherapy. Moreover, immunotherapy can reverse chemoresistance and compensate for the reduced specificity of chemotherapy that may arise due to long-term treatment in patients^139^^,^^140^. Consequently, chemo-immunotherapy is becoming popular, and the use of SDDSs enables the programmable temporal release as well as spatial distribution of chemotherapeutic and immunotherapeutic drugs, thereby achieving amplified treatment efficacy.

#### 3.1.1 Immune enhancement (direct activation of immune system)

The most commonly used modality in treatment is to deliver cytotoxic and immunotherapeutic agents into tumor cells and TME, respectively. For instance, the HA-DOX/PHis/R848 nanoparticles (NPs) were designed to improve immunomodulatory effect and provide targeted cytotoxicity against tumor cells^128^. The conversion of poly-histidine (PHis) from hydrophobic to hydrophilic characteristics aids in the disintegration of NPs and promotes the release of immunomodulator R848 in the TME to activate immune cells. Subsequently, hyaluronic acid (HA) delivered the cytotoxic doxorubicin (DOX) into tumor cells to directly destroy them. Another carrier, MPH-NP@A, which also incorporated HA, was engineered by combining cross-linkers of matrix metalloproteinase-9 (MMP9)-responsive peptides with pre-produced acid-sensitive PH-NPs (formed by aPD-L1 and HA-acetal-paclitaxel (HA-ace-PTX(SH))^113^. In a 4T1 mouse model, this carrier markedly enhanced anti-tumor activity *via* asynchronous effects of chemotherapeutic agents and ICI. In detail, MPH-NP@A remains stable during circulation. However, upon entering the TME, where MMP9 is highly expressed, it is cleaved and aPD-L1 is released. On the tumor cell surface, the loosened PH-NPs were recognized and endocytosed by CD44, and then degraded in the progressively acidified endosomes to release PTX. This multisite-specific release behavior fully exploited the cytotoxic effect of PTX and enhanced the T cell immune enhancing effect of aPD-L1. As a consequence, protein stability and pharmacokinetic (PK) parameters are improved, and irAEs are reduced. Similarly, Su et al.^129^ constructed pH and MMP dual-sensitive micelles for enhanced chemo-immunotherapy, with the drug difference being the replacement of aPD-L1 with aPD-1. Moreover, several studies have demonstrated that chemotherapeutic agents in combination therapy upregulate the expression of immune checkpoints in tumor cells, which further justifies ICI combination therapy^141^.

Aside from directly targeting cytotoxic T cells, agents targeting APCs are also popular because APCs play an crucial role in the direct activation of anti-tumor immunity, with dendritic cells (DCs) being regarded as the immune response’s master regulators due to their ability to initiate all antigen-specific immune responses^142^. Therefore, a novel strategy named as “relay delivery” was put forward to amplify the effects of chemo-immunotherapy through targeting tumor cells and DCs^130^. This strategy facilitates the delivery of the drug to the AS1441 aptamer (Apt) located in the nucleus of cancer cells by relying on the extracellular dissociation of human high-density lipoprotein (HDL). The pre-formed HDL NPs were encapsulated by the AS1411-CpG fusion motif while DOX was inserted into the CG base pair to obtain imHDL/Apt-CpG-Dox. When the nanodrug reaches the TME, the dissociation of the HDL structure and release of Apt-CpG-Dox into the interstitial space occurred as a result of HDL recognition. The coupling of AS1441 with nucleophosmin allowed Apt-CpG-Dox to enter the cell and translocate to the nucleus. Subsequently, the CpG motif released from dead cancer cells is captured by infiltrating APCs, thereby activating toll-like receptor 9 (TLR9). Activated DCs would secrete cytokines to facilitate antigen presentation and coordination of the immune cascade.

Based on research conducted over the last few decades, novel local cancer vaccines have been developed that exhibit greater therapeutic potential than conventional predefined vaccines by utilizing autologous tumors as the source of antigens^143^. Morever, adequate activation of immune cells in tumor draining lymph nodes (TdLNs) during vaccination to trigger anti-tumor immunity is essential to generate robust adaptive immunity^144^. However, structurally homogeneous delivery vectors failed to meet the requirements of the complicated immune activation process, leading to inefficient accumulation of tumor antigens in TdLNs. Considering these obstacles, Zhang et al.^124^ designed a nanodrug (PIAN) that can dynamically adapt to specific environments. PIAN was created through the formation of a host–guest interaction between CpG/polyamidoamine-thioketal-adamantane (CpG/PAMAM-TK-Ad) and poly-[(*N*-2-hydroxyethyl)-aspartamide]-Pt (IV)/*β*-cyclodextrin (PPCD). After intravenous injection, PIAN accumulated in tumor tissues due to the enhanced permeability and retention (EPR) effect. Elevated ROS level in TME cause PIAN to dissociate, releasing polyethylene glycol (PEG), PPCD and positively charged CpG/PAMAM along with it. PPCD can lead to tumor cell death and antigen release, then CpG/PAMAM may capture antigen and transfer it to TdLNs. This novel vaccine cured 40% of mice with a colorectal cancer model by combination therapy with aPD-L1. Overall, PIAN provided a new framework for the design of sequential programmable nanomedicine.

Even though chemo-immunotherapy kills the majority of tumor, a specific type of cancer cells called cancer stem cells (CSC) can still cause tumor metastasis and recurrence in TME^145^^,^^146^. To overcome chemo-immunotherapy resistance mediated by CSC^147^, SDDSs can be loaded with three different drugs simultaneously in comparison to the classic two-drug therapy. Lang et al.^131^ constructed a micelle-liposome bilayer structured enzyme/PH dual responsive nanocarrier PM@THL to deliver HY19991 (ICI), thioridazine (THZ, an anti-CSC agent) and PTX. The initial carrier was stable in circulation, but in TME, the outer layer was stripped by MMP, and then released THZ and ICI. The inner layer’s micelles were tiny and released PTX in the endosomal acidic compartment after uptake by tumor cells. In a metastatic breast cancer model using MCF-7 cells, the drug-loaded PM@THL exhibited remarkable anti-cancer effects with the tumor inhibition rate of 93.5% and lung metastasis inhibition rate of 97.6%. It also reduced the proportion of CSC and enhanced T cell infiltration in tumor tissues. Spatiotemporally-tuned nanodevice based cocktail therapy will provide a promising strategy for cancer treatment.

#### 3.1.2 Immune normalization (attenuating the tumor immunosuppression & remodeling the TME)

The second therapeutic direction is concentrated on reprogramming the tumor immune microenvironment. The TME is populated by a variety of immunosuppressive cells, including myeloid-derived suppressor cells, TAMs, and cancer-associated fibroblasts. Immunosuppressive cells exert a significant inhibitory effect on the infiltration and function of cytotoxic lymphocytes, as well as secrete pro-tumor cytokines that promote ongoing tumor growth^148^. SDDSs enable the selective delivery of multiple therapeutics to immunosuppressive cells and tumors, which allows for synergistic multimodal treatment.

Cancer-associated fibroblasts (CAFs), that are usually regarded as one of the main contributors to immunosuppressive TME, are responsible for tumor formation, invasion, and metastasis^81^. CAFs are derived from normal fibroblasts (NAFs) upon activation by TGF-*β*, and tumor cells secrete high levels of TGF-*β*, implying a reciprocal relationship between CAFs and TGF-*β*. It has been found that TGF-*β* receptor inhibitors can suppress the formation of CAFs^149^, which may be a new target to improve the TME. Zhang et al.^118^ introduced a TME-responsive sequential nanoparticle-based strategy named NLC/H (D + F + S), which integrates three mechanisms: ferroptosis, ICD, and TME reprogramming. Ferroptosis is a form of programmed cell death characterized by accumulation of ferric ions, lipid peroxidation, and elevated ROS levels^150^. DOX causes ferroptosis by activating nicotinamide adenine dinucleotide phosphate oxidases (NOXs), which raise the H_2_O_2_ levels in tumor cells^151^. Ferrocene (Fc) is an exogenous ferroptosis inducer that elevates intracellular ROS levels through the Fenton reaction^152^, and high levels of ROS-mediated cell death can in turn induce ICD^91^. DOX and Fc were loaded into cationic lipid NPs, and then *β*-cyclodextrins grafted heparin (Hep-*β*-CD) loaded with the TGF-*β* receptor blocker SB431542 were wrapped around these NPs to form NLC/H (D + F + S). As the carrier arrived at the TME, it was stripped of its outer coating by high levels of heparanase (HSPE) in the TME, which then released SB to limit TGF-*β* secretion and CAFs formation. Meanwhile, charge inversion occurred in the carrier to promote tumor cell internalization. DOX and Fc were released intracellularly in parallel, boosting the tumoricidal effect of DOX *via* a hybrid apoptosis/ferroptosis mechanism. Additionally, ferroptosis further enhanced the ICD caused by DOX. Moreover, elevated ROS expression decreased MMP9 expression in tumor cells and synergized with extracellular SB for anti-metastasis. In brief, NLC/H (D + F + S) utilized a sequential approach that synergistically harnessed multiple mechanisms to achieve a potent anti-tumor outcome.

**Figure 3:**
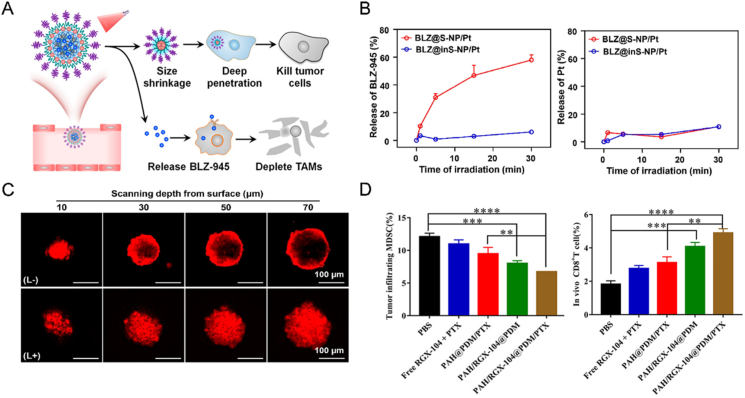
SDDS-based chemo-immunotherapy strategies for immune normalization. (A) Under 660 nm laser irradiation, BLZ-945 and Pt(IV) were sequentially delivered to M2-like TAM and tumor cells, resulting in combination chemo-immunotherapy for synergistic anti-tumor effects. (B) Light irradiation effectively triggered the release of BLZ-945 from BLZS-NP/Pt but had no effect on the release of Pt(IV) prodrug. (C) CLSM observed that light irradiation promoted the deep penetration of BLZS-NP/Pt into tumor multicellular spheres (MCSs). Reprinted with the permission from Ref. 115. Copyright © 2021 American Chemical Society. (D) Flow cytometry was used to detect the immune status of the TME in each treatment group. Reprinted with the permission from Ref. 112. Copyright © 2020 Elsevier.

TAMs can promote malignancy by facilitating immune escape and tumor progression. Therefore, elimination or reprogramming of TAMs is crucial for achieving immune normalization^153^. Additionally, the anti-tumor efficacy of SDDSs relies heavily on the carrier’s capacity to selectively deliver drugs to cancer cells or the TME. The laser-responsive scalable nanoplatform BLZ@S-NP/Pt was designed to target specifically TAMs and tumor cells (Fig. 3A)^115^. Besides, it possesses the capability to penetrate deeply into tumors. In detail, the initial carrier size was adjusted to 70 nm, which facilitated preferential aggregation of tumor in the perivascular region. Artificial irradiation using a 660 nm laser disrupted the core of the platform and triggered the rapid release of BLZ945, which inhibits CSF-1R (the surface receptor of TAMs) (Fig. 3B). Subsequently, the carrier shrank into smaller particles (∼30 nm) that were coupled to Pt (IV) prodrugs and further entered into the tumor mesenchyme, which in turn delivered the chemotherapeutic agent to the entire tumor (Fig. 3C). This photoactivated platform successfully overcame the spatial distribution barrier of various cells, depleted TAMs, and killed cancer cells. Ultimately, a synergistic anti-cancer effect of inhibiting tumor growth, preventing metastasis, and prolonging survival was achieved in multiple tumor models.

MDSCs are one of the major components in the immunosuppressive TEM and employ multiple mechanisms to suppress intrinsic and adaptive immunity, including ROS generation to promote oxidative stress, activation and proliferation of Treg, inhibition of lymphocyte trafficking, and depletion of lymphocyte nutrients^154^^,^^155^. MDSCs also support tumor activity and metastasis by secreting a variety of immunosuppressive molecules such as VEGF, MMP, TGF-*β*, and IL-10^156^. Therefore, depletion of MDSCs is an effective strategy to alleviate immunosuppression. Liver X receptor (LXR*α*/*β*) is a nuclear hormone receptor that stimulates tumor angiogenesis and metastasis through inducing transcriptional activation of apolipoprotein E (ApoE), a protein secreted by stromal cells (*e.g.*, endothelial cells, macrophages) in TME^157^, ^158^, ^159^, ^160^. Considering the recent report on the inhibitory effect of LXR/ApoE axis activation on MDSC activity, RGX-104, an LXR*β* agonist, can effectively decrease the MDSC level and impede tumor growth^161^. Wan et al. engineered dual pH-responsive conjugated micelles (PAH/RGX-104@PDM/PTX) loaded with RGX-104 and PTX to perform chemo-immunotherapy by successive depletion of MDSCs and cancer cells^112^. Upon reaching the acidic TME, the carrier disintegrated, releasing RGX-104 in the vicinity of the tumor vasculature, a region characterized by stromal cell aggregation that fosters tumor progression and metastasis. RGX-104 was preferentially taken up by macrophages, activating the LXR/ApoE axis and attenuating the activity of MDSCs. As a result, the remaining small-sized particles were able to penetrate deeper into the tumor and release PTX in the acidic endosomes of cancer cells. Eventually, the depletion of MDSCs weakened TME immunosuppression and augmented the tumoricidal effect of tumor infiltrating lymphocytes (TILs) (Fig. 3D). Furthermore, the anti-tumor effect of PTX was also improved.

### 3.2 Combination strategy of photo-immunotherapy with biomaterial-based SDDSs

Cancer phototherapy is a treatment modality that harnesses the biological effects produced by nanomaterials and mediated by external physical light sources. It has garnered significant attention in the realm of fundamental research^162^. In this context, nanomaterials function as both the carrier and the primary component of the treatment. Phototherapy is divided into photodynamic therapy (PDT) and photothermal therapy (PTT). To treat cancers, PDT uses light-mediated photosensitizers to generate singlet oxygen and reactive oxygen radicals to treat tumors, whereas PTT employs near-infrared light to mediate the thermal action of light-absorbing substances in order to locally heat and ablate tumors^163^. A number of studies have shown that both PDT and PTT are able to elicit immune responses through diverse mechanisms, including ICD, enhancement of immune cell activity, and remodeling of the TME^163^. Thus, the integration of immunotherapy and phototherapy gives rise to a novel anti-cancer therapeutic technique known as photo-immunotherapy (PIT). This synergistic treatment approach not only enhances the effectiveness of both therapies but also surmounts their inherent limitations. SDDSs precisely deliver drugs and photosensitizers/photothermal agents to designated locations of tumor tissue, hence reducing side effects and amplifying anti-tumor immune responses. Furthermore, such engineered carriers with internal design and external energy input to modulate and control the sequential process can provide a more diverse supply of therapeutic agents and generate greater anti-cancer effects in local TME.

#### 3.2.1 Photodynamic-immunotherapy with biomaterial-based SDDSs

Tumor photodynamic therapy utilizes laser light of specific wavelengths to activate photosensitizing drugs that are aggregated in tumor tissues. Subsequently, a photochemical reaction occurs with the participation of oxygen in biological tissues to produce oxygen radicals and singlet oxygen, which in turn causes tumor tissue destruction and apoptosis^164^. Traditional PDT has been considered as an effective strategy for tumor treatment, however, this method has some limitations. From one aspect, the unsuitable NPs size normally leads to the poor penetration of carriers or the paradoxical size-dependent EPR effects. In addition, it is difficult for treatment by single agent to have a sufficient impact^165^^,^^166^. Therefore, photodynamic-immunotherapy was put forward to achieve an ideal result. Yu et al.^123^ created sequentially responsive size-reducible biomimetic NPs. Small-sized cationic gold nanoclusters (CAuNCs) were wrapped by HA to form optimal-sized initial NPs CAuNCs@HA. Since CD47 on red blood cell (RBC) membranes prevented macrophage clearance^167^, the authors wrapped CAuNCs@HA in RBC membranes to bypass the mononuclear-phagocyte system (MPS). When CAuNCs@HA arrived at the TME, they were degraded to CAuNCs by high levels of hyaluronidase, lowering particle size and releasing dPPA (PD-1/PD-L1 axis blocker). PDT under radiation raised the intracellular ROS level by absorption of CAuNCs by tumor cells. Following that, cinnamaldehyde and thioacetal-based PTX dimer precursor (PXTK) interacted with ROS to produce PTX monomers, and this hydrolysis process stimulated mitochondrial production of ROS. *in vitro* and *in vivo*, this combination therapy was shown to enhance cell-mediated immunity (CD4^+^, CD8^+^ T cells and NK cells), increase the secretion of anti-tumor cytokines (tumor necrosis factor-*α* (TNF-*α*), IL-12) and demonstrate exciting tumor suppressive and anti-metastatic effects in a 4T1 model of breast cancer. Similarly, Feng et al.^119^ developed MB@MSP, a TME-responsive nanomedicine for PDT combined with ICB therapy. MB@MSP achieved shrinkage and charge reversal through MMP-2 and GSH in TME, further enhancing the accumulation of NPs at the tumor site. This photodynamic-immunotherapy showed potent anti-tumor immune effects in both lung cancer local and metastatic tumors.

**Figure 4:**
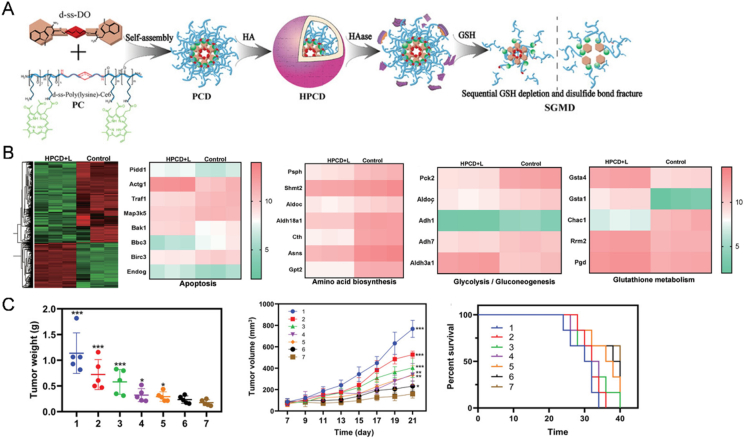
SDDS-based photodynamic–immunotherapy for combination tumor treatment. (A) Schematic illustration of the synthesis and action of HPCD loaded with IDO inhibitor dimer (d-ss-DO). (B) RNA sequencing of treated tumors showed detailed functional genomic heat maps related to apoptosis, amino acid biosynthesis, glycolysis/gluconeogenesis, and glutathione metabolism in HPCD + L treated group. (C) Tumor growth curve, tumor weight at the end of the experiment monitoring period, and survival curve of tumor-bearing mice in different groups. Groups 1–7 represented PBS, HPCD, HPC + L, HPLCM + L, HPCM + L, HPCD + L, and HPCD + L + anti-PD-L1, respectively. Reprinted with the permission from Ref. 132. Copyright © 2022 Wiley-VCH.

It has been proposed that accumulation of ROS can induce immunotherapy associated with palliation of suppressive TME^168^, but the extent of this therapy is insufficient to exert a powerful therapeutic effect. If immune checkpoint-mediated immune escape can be eliminated, ROS-activated immunotherapy has the potential for curing tumors. To achieve high levels of ROS accumulation, PDT and GSH depletion can be combined, as both disrupt redox homeostasis and induce an imbalance in cellular homeostasis. Lei et al.^132^ used sequential amino acid metabolism disruption (SAAMD) to disturb tumor cell homeostasis and improve photodynamic-immunotherapy. The polymeric NPs loaded with the indoleamine-2,3-dioxygenase inhibitor (IDOi) dimer (d-ss-DO) were assembled through a disulfide-bridged copolymer tethered to the photosensitizer Ce6, which acted as a glutathione peroxidase enzyme to consume GSH. At the same time, disulfide bond breakage led to a loosening of the NPs structure, which facilitated the release of d-ss-DO. The process of dimer catabolism was necessary for further GSH consumption, so these two processes constituted a sequential GSH metabolic disorder (SGMD) (Fig. 4A). Subsequently, monomeric IDOi blocked the tumor escape mechanism (tryptophan metabolic process) and synergized with accumulated ROS to alleviate the inhibitory TME. Furthermore, the outermost HA of NPs can contribute to an increase in NPs accumulation in cancer tissues, boosting ROS production and SAAMD (GSH/Trp). RNA sequencing significantly supports the conclusion that HPCD can sequentially disrupt GSH and amino acid metabolism, cut off material and energy supplies essential for tumor proliferation, and induce apoptosis in direct PDT-induced ROS-based anti-tumor (Fig. 4B). This strategy greatly enhanced photodynamically induced immunotherapeutic outcomes against breast cancer and melanoma, especially when combined with PD-L1 blockade (Fig. 4C).

In another work, Su et al. also concluded that the immune response induced by PDT alone was insufficient for anti-tumor effects, so they developed a hierarchical carrier for the delivery of the photosensitizers Zinc phthalocyanine (ZnPc) and aPD-L1 to improve photodynamic-immunotherapy^122^. First, MPc was formed by loading ZnPc into polymeric micelles, and then immobilizing MMP-sensitive peptides coupled to aPD-L1 and sheddable pH-sensitive PEG corona on the surface to generate sAMPc. The difference in spatial distribution (TME/intracellular) of ICI and ZnPc was achieved by structural alternations in the nanocarriers, which were attributed to particle size reduction based on pH response and charge inversion based on MMP response. Several studies in this area demonstrate that photoactivated ROS generation and subsequent ROS-triggered local release of ROS-sensitive and PDT-capable nanodrugs can be sequentially used for photodynamic-immunotherapy. Moreover, these therapeutic agents can establish a spatiotemporal relationship to provide synergistic effects.

#### 3.2.2 Photothermal-immunotherapy with biomaterial-based SDDSs

PTT is a non-invasive local treatment with irradiation controllability that can be applied to enhance the efficacy of combination anti-cancer therapy in SDDSs. It has been pointed out that markers of ICD were upregulated after PTT to promote DC maturation. Mild PTT, on the other hand, induced upregulation of PD-L1 on the surface of cancer cells and enhanced tumor self-protection^169^. Therefore, it is reasonable to intervene with the cancer cells using photothermal-immunotherapy. In addition, photothermal agents, as the crux of PTT, should have the following characteristics: (1) Strong near-infrared light absorption and photothermal conversion efficiency. (2) Reasonable biological behavior and certain tumor enrichment ability. (3) Good biosafety. The rapid development of the field of nanomaterials has expanded the range of options for PTT photothermal agents.

Recently, Xu et al.^170^ reported a new type of 2D material—few-layer bismuthene—that has higher photothermal conversion efficiency compared with conventional photothermal agents. More importantly, these nanomaterials have a large specific surface area and a folded honeycomb 2D structure, along with superior biocompatibility and low biotoxicity. These reasons point to bismuthene’s potential as a drug delivery platform. Guo et al.^133^ used bismuthene-based dual-responsive nanoplatform for successive PD-L1 blockade and PTT. PEGylated polyethyleneimine (PEI) wrapped bismuthene to form Bi@PP while adsorbing siPD-L1 by electrostatic force. When this “one-for-all” carrier was exposed to the acidic environment of TME, the rapid dissociation of PEG led to a reduction in carrier size and charge reversal. The remaining carriers were internalized by the tumor cells and the amino protonation of PEI induced proton sponge effect to promote endosome escape. Meanwhile, photoinitiation not only enforced PTT but also accelerated endosome escape and triggered siRNA release. It was surprising to find that mild PTT was able to elicit enhanced pathological permeability and retention (EPPR) effect, showing the great advantage of photothermal-immunotherapy in both primary and metastatic tumors in 4T1 model. Besides, biosafety evaluation demonstrated effective clearance of Bi@PP by both the intestine and kidney, avoiding potential toxicity risks.

Apart from the classic PD-1/PD-L1 axis, IDOi-based immunotherapies can also be combined with PTT^171^^,^^172^. A recent phase III clinical failure of epacadostat (IDO inhibitor) in combination with keytruda (PD-1 inhibitor) was very frustrating for researchers. The failure was attributed to low bioavailability and inadequate intra-tumor drug exposure^173^. If the dense extracellular cell matrix (ECM) and high interstitial fluid pressure of solid tumors can be overcome, drug penetration and tumor cells death can be maximized. Liu et al.^116^ used dual-responsive self-assembled nanocarriers delivering IDOi and the photosensitizer indocyanine green (ICG) in combination with PD-L1 blockade to develop successful photothermal-immunotherapy. The bilayer NPs pro-drug (mPEG-Pep-IDOi/ICG) was formed *via* electrostatic interactions and *π*–*π* stacking interactions between self-assembly of PEGylated epacadostat and amphiphilic photosensitizers. When NPs accumulated in the TME through the EPR effect, the PEG layer was stripped by MMP2, resulting in altered molecular interactions. Subsequently, the core layer of NPs, *i.e.*, smaller IDOi/ICG aggregates, were liberated from the large size pro-drug to improve deep penetration. Finally, NIR laser irradiation triggered PTT to kill tumor cells and released antigens that promoted the maturation of DCs, followed by IDOi released from dead tumor cells, further improving T cell responses in TME. This work provided a novel delivery method for photothermal–immunotherapy and IDO inhibition.

### 3.3 Combination strategy of gene-immunotherapy with biomaterial-based SDDSs

Gene-immunotherapy was defined as the integration of transgenic delivery or gene regulators to compensate or correct mutated genes in tumor cells directly *in vivo*, or to genetically modulate immune cells to enhance anti-tumor immune responses for cancer treatment^174^^,^^175^. Among these approaches, breakthroughs in RNA interference (RNAi) and mRNA technology are leading to their practicalization. The world’s first siRNA drug, ONPATTRO (Patisiran), was approved for marketing by the FDA in 2018^176^. This is a momentous milestone in the field of siRNA delivery, and it has paved the path for future siRNA therapeutics. The greatest advantage of this drug class is its target specificity, which enables precision and personalized therapy. Yet, the site of action for siRNAs must be intracellular, requiring them to overcome multiple extra/intracellular barriers such as nucleases, MPS, permeation barriers, cellular exocytosis, endosomal escape, and others^177^^,^^178^. Therefore, the stability and pharmacokinetic behavior of siRNA largely influence the efficacy of siRNA therapy^179^. Obviously, SDDSs-based gene-immunotherapy can amplify the effectiveness of cancer therapy and meanwhile reduce off-target effects *via* spatiotemporal delivery of siRNA in a programmed manner.

Tumor therapy is gradually transitioning from the era of pre-genomic cytotoxic drug therapy to the new era of post-genomic targeted therapy. Gene therapy has the effect of specifically targeting certain tumor gene mutations to achieve precise killing, while different tumor patients have different immune escape gene mutations. Therefore, gene-immunotherapy can be customized to target various refractory tumors to restore the body’s anti-tumor immunity. Triple-negative breast cancer (TNBC) as an intractable tumor, has been focused in the field of gene - immunotherapy due to the high expression of PD-L1 and CD155^180^. CD155 functions as a ligand for the co-stimulatory receptor DNAM-1 and the co-inhibitory receptor TIGHT/CD96 on CD8^+^ T cells^181^. Unfortunately, the latter receptor has a higher affinity for CD155 than the former one^182^, and this competitive binding causes a reduced anti-tumor effect of CD8^+^ TILs and tumor immune escape^183^. It has been reported that both PD-1/PD-L1 and CD155/TIGHT axes were negative regulators of DNAM-1 expression^184^, suggesting that combining PD-1/PD-L1 axis blockade with TIGHT/CD96 blockade may optimize anti-tumor immunity. Chen et al. discovered that PD-1 and DNAM-1 expression in CD8^+^ TILs occurred earlier, but CD96/TIGHT expression occurred later^38^, implying that early CD155 supported anti-tumor immunity while late siRNA blocked the immune escape of CD155. Through sequential delivery of siRNA and aPD-L1 to asynchronously block CD155 and the PD-1/PD-L1 axis, they successfully inhibited the growth and metastasis of TNBC^38^. P/PEAL_siCD155_ NPs were produced by self-assembly of positively charged mPEG-PLGA-PLL with negatively charged siCD155 and aPD-L1. aPD-L1 interacted with PD-L1 immediately after NPs contacted tumor cells, leading to ICB. Simultaneously, tumor cells took up NPs through receptor-mediated endocytosis, and siCD155 mediated silencing occurred afterwards. This carrier greatly boosted the anti-tumor immunity dominated by CD8^+^ TILs, enabling precise targeting of cell surface receptors and intracellular genes in a spatiotemporal manner.

Currently, gene-immunotherapy as a standalone treatment is not effective enough, which may be related to the fact that the window of effectiveness concentration is not wide enough^185^. Therefore, some studies have combined it with photo-immunotherapy or chemo-immunotherapy to exert synergistic effects. The Bi@PP-based photo-immunotherapy mentioned in section 3.2.2 employed siRNA to silence the PD-L1 gene in tumor cells. Similarly, Yi et al.^134^ used the peptide-based siRNA micelle complex P^A7R^@siPD-L1 to reverse vascular normalization-mediated immunosuppressive TME and enhance photo-immunotherapy synergistically. The micelles were disassembled by MMP2 upon reaching TME, releasing the anti-angiogenic peptide A7R and PEG-R_9_K (Ce6)-LLGPLG@siPDL-1. The former peptide then targeted vascular endothelial growth factor receptor-2 (VEGFR-2) and neural adhesion protein-1 (NRP-1) on tumor endothelial cells to normalize tumor vasculature, alleviate hypoxia, and promote immune cell infiltration. Meanwhile, the latter one entered tumor cells with the assistance of the cell-penetrating peptide R_9_, followed by manually controlled laser irradiation, which triggered PTT (increased ROS and induction of ICD) and lysosomal rupture due to elevated ROS levels. The siPD-L1 released from the lysosomes diffused into the cytoplasm and silenced the PD-L1 gene to reduce immune tolerance. The key implication of this work was the availability of a universal delivery carrier that can be loaded with siRNAs against different targets for the synergistic treatment of various malignancies.

**Figure 5:**
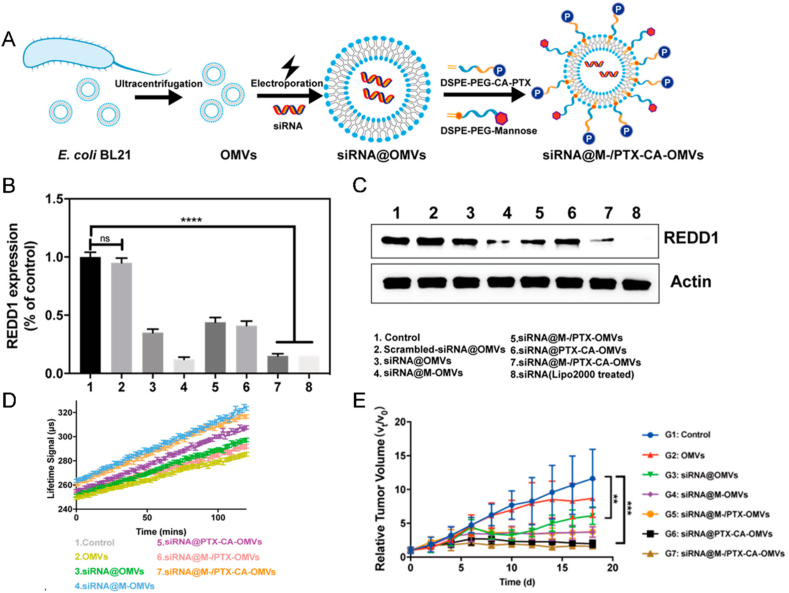
SDDS-based gene-immunotherapy for combination tumor treatment. (A) Preparation of siRNA@M-/PTX-CA-OMVs. (B, C) Redd1 expression in Raw264.7 cells after IL-4 pretreatment was detected by RT-qPCR and Western blot, and siRNA@M-/PTX-CA-OMVs were comparable to the positive control (Lipo2000). (D) Lifetime profiles of glycolysis assay of RAW 264.7 cells treated with different treatment groups. (E) Tumor volume growth curves of different groups in mice carrying TNBC. Reprinted with the permission from Ref. 136. Copyright © 2021 American Chemical Society.

In combination with chemotherapy, siRNA-based immunotherapies can disrupt the tumor metabolic microenvironment and modify immunosuppressive TME by repolarizing TAMs^186^, ^187^, ^188^. Specifically, glycolysis inhibition suppresses tumors, but also renders M2-like TAMs resistant to repolarization^189^. Response1 (Redd1) acts as a negative regulator of the mechanistic target of rapamycin (mTOR), a crucial molecule involved in glycolysis^190^^,^^191^. By downregulating Redd1 in TAMs, the metabolic phenotype can be altered through increased glycolysis levels, despite the undesirability of elevated glycolysis in cancer cells^192^. Hence, differentiated metabolic modulation techniques must be developed for M2-like TAMs and malignant cells to achieve synergistic therapeutic effects. Guo et al. developed pH-responsive bacterial outer membrane vesicles (OMVs) for specific targeting of distinct cell populations within the TME^136^. OMVs were highly immunogenic, easily recognized and phagocytosed by macrophages, and can further enhance TAMs targeting by mannose modifications. siRedd1 was loaded into OMVs by electroporation (Fig. 5A). Since the upregulation of Redd1 in macrophages was associated with metabolic stress (hypoxia) in cancer cells, PTX-mediated cancer cells death could be employed to cut off this intercellular metabolic communication. The pH-sensitive linker *cis*-aconitate (CA) was then introduced between DSPE-PEG and PTX and inserted into the phospholipid bilayer of OMVs to form siRNA@M-/PTX-CA-OMVs. After the engineered OMVs were circulated to tumor tissue, the acidic TME triggered the release of PTX. Subsequently, the remaining portion of the carrier was internalized by M2 TAMs. *in vitro* cellular experiments revealed that the silencing of Redd1 led to a decrease in glycolysis levels in RAW 264.7 cells (Fig. 5B‒D). This strategy showed remarkable TAM repolarization, immune activation, TME reconstitution, and tumor suppression in TNBC models (Fig. 5E). Moreover, the utilization of OMVs offered novel insights into platforms for sequentially targeting multiple cell populations.

## 4 Application of biomaterial-based SDDSs for local drug delivery

Local administration greatly increased drug concentrations at the focal site, avoided transport in the circulation, reduced distribution in non-target tissues, and decreased the occurrence of irAEs. The most commonly used local drug delivery platform is the gel scaffold^193^, ^194^, ^195^, however, alternative carriers such as micelles^196^^,^^197^, nanoemulsions^198^, and micro/nanoparticles^199^, ^200^, ^201^, are also used. Mechanisms for achieving sequential drug release include affinity-based approaches, such as drug-material electrostatic interactions^202^, ^203^, ^204^, and severable covalent linkages^205^^,^^206^; diffusion-dependent methods influenced by drug molecular weight^194^^,^^207^^,^^208^, and mesh/cargo pore size^209^, ^210^, ^211^; and physical encapsulation-based techniques like nanoparticle-gel hybrid systems^195^^,^^212^, and shell–core structures^198^^,^^199^^,^^213^. Considering some patients are not suitable surgical candidates for clinical practice, this section is divided into two subsections: post-tumor resection treatment and non-surgical treatment. Furthermore, diverse strategies for local sequential drug delivery system are also summarized and discussed (Table 2^193^, ^194^, ^195^, ^196^, ^197^, ^198^, ^199^^,^^202^^,^^204^^,^^205^^,^^208^^,^^209^^,^^212^, ^213^, ^214^, ^215^).

**Table 2:** Recent advances in SDDSs for local drug delivery in tumor immunotherapy.

Postoperative or intra/peri-tumoral injection	Mechanism/method	Therapeutics	Material	Immune effect	Ref.
Postoperative	Molecular weight	CTX + aPD-L1	Fibrin gel	ICD synergized with ICB to enhance CMI	193
Postoperative	Electrostatic interaction	DOX-Lip + aPD-L1	MMP-sensitive hydrogels (CS-MMP-Pul)	ICD synergized with ICB to enhance CMI	202
Postoperative	Multi-stage structure	MIT + CXCL10 + siIDO1	Oligopeptide hydrogel	Enhanced CMI and attenuated immunosuppression	214
Postoperative	Size of mesh/cargo	Anti-PD-L1-conjugated platelets + CAR-T cells	HA hydrogel	Improved TME and relieved immunosuppression	209
Postoperative	Shell‒core structure	Sorafenib-GO-NPs + aCD47	Mixture of SPC and GDO	Repolarization of TAMs for improved TME	213
Postoperative	Electrostatic interaction	Cyc-Lip + aCD47	Redox-sensitive hydrogel (CS-Pul)	Repolarization of TAMs for improved TME	204
Postoperative	Shell‒core structure	CaCO3 + aCD47	Fibrin gel	Repolarization of TAMs and enhanced CMI	215
Postoperative	Multi-stage structure	DOX + R848	Collagen-HA hydrogels	Induced ICD and repolarized suppressor cells	195
Peritumoral injection	Molecular weight	GEM + aPD-L1	ROS-responsive hydrogel (PVA + TSPBA)	Repolarization of suppressor cells and improved TME	194
Intratumoral injection	Cleavable covalent connection	aPD-L1 + D-1MT	ROS-degradable peptide-gel (P(Me-D-1MT)-PEG-P(Me-D-1MT))	Enhanced CMI and alleviation of inhibitory TME	205
Peritumoral injection	Integration into cross-linked networks	GEM + D-1MT	Hybrid hydrogels (CS-SH + F127-MA micelles)	Induced ICD to enhance CMI and alleviation of inhibitory TME	196
Peritumoral injection	Multi-stage structure	REG + LY3200882	Temperature-sensitive hydrogel (mPEG-*b*-PAla)	Enhancement of CMI and mitigation of suppressive TME	212
Peritumoral injection	Molecular weight	CDDP + IL-15	Temperature-sensitive hydrogel (mPEG-*b*-PELG)	Enhancement of CMI	208
Peritumoral injection	pH ultra-sensitive platform	DOX + IMDQ	Nanoparticles (PEG-*b*-P (C7A-r-ss-DOX) and PEG-*b*-P (iPDA-r-IMDQ))	Induction of ICD and strong activation of CMI *via* SLNs	197
Intratumoral injection	Shell‒core structure	aCD47 + aPD-1	ROS-sensitive protein complex (albumin and IgG)	Reduction of Treg cell and polarized TAMs to improved TME	199
Intratumoral injection	Shell‒core structure	DOX + HY	pH-responsive pickering nano-emulsion	ICD synergized with ICB to enhance CMI	198

CTX, cyclophosphamide; DOX, doxorubicin; TME, tumor microenvironment; MIT, mitoxantrone; CXCL10, chemokine ligand 10; CMI, cell-mediated immunity; CS, chitosan; MMP, matrix metalloproteinase; Pul, pullulan; HA, hyaluronic acid; CAR, chimeric antigen receptor; SPC, phosphatidylcholine; GDO, soybean glycerol dioleate; GO-NPs, graphene oxide-nanoparticles; Cyc-lip, cyclopamine liposome; ICD, immunogenic cell death; aPD-1, anti-programmed cell death-1; aPD-L1, anti-programmed cell death ligand-1; GEM, gemcitabine; ROS, reactive oxygen species; PVA, polyvinyl alcohol; TSPBA, *N*^1^-(4-boronobenzyl)-*N*^3^-(4-boronophenyl)-*N*^1^,*N*^1^,*N*^3^,*N*^3^-tetramethylpropane-1,3-diaminium; REG, regorafenib; CDDP, cisplatin; IL-15, interleukin-15; IMDQ, Imidazoquinoline; PEG, polyethylene glycol; aCD47, anti-CD47 antibody; ICB, immune checkpoint blockade; TAMs, tumor-associated macrophages; siRNA, small interfering RNA; IgG, immunoglobulin G.

### 4.1 Local drug delivery for post-tumor resection treatment

Surgical resection is the predominant clinical approach for solid tumors^216^. Although surgery can show progression over time, patients remain vulnerable to tumor recurrence and metastasis^217^^,^^218^, which can be explained by Stephen Paget’s “seed and soil” theory. According to this perspective, cancer cells are analogous to seeds, while the tumor-friendly milieu in the body is likened to soil. The aggressive growth pattern of tumors can result in incomplete surgical resection of solid tumors, including highly infiltrative tumors that pose inherent challenges for complete removal (*e.g.*, brain glioma). As a result, residual tumor cells may be present at the surgical margins as well as in the circulation. Moreover, the systemic immunosuppression during the perioperative period creates a conducive environment for the revival of dormant seeds^219^^,^^220^. Once the conditions are favorable, the tumor will promptly recur and metastasize. Fortunately, the utilization of SDDSs can successively eradicate the residual tumor cells and optimize the TME, thereby impeding the progression of deadly tumors.

#### 4.1.1 Killing residual seeds and amplifying the immune effect by immune enhancement

ICD is a form of regulatory cell death (RCD). This process is accompanied by the secretion or release of damage molecule-associated patterns (DAMPs) including calreticulin (CRT), adenosine triphosphate (ATP), and high migration histone 1 (HMBG1)^91^. Subsequently, these DAMPs serve as immune adjuvants to activate cell-mediated immunity. Several chemotherapeutic drugs that kill tumor seeds, such as DOX, cyclophosphamide (CTX), and oxaliplatin, have been proven in studies to be effective ICD inducers^221^^,^^222^. When ICD is combined with immune checkpoint blockade (ICB) *via* SDDSs, the latter functions as an immune amplifier against tumors. Zhang et al. created a fibrin gel-based scaffold (FDA-approved for human use) for loading CTX and aPD-L1 to prevent recurrence of breast and ovarian cancer after surgery^193^. Due to the difference in drug molecular weight, CTX was released more rapidly from the gel to kill cancer cells and induce ICD effects. Following that, the phenotype of TME was stimulated to be immunogenic, which favors lymphocyte infiltration and cell-mediated immunity. Slower-releasing aPD-L1 significantly enhanced the locally initiated anti-tumor response *via* ICB. SDDSs exploited variations in the release kinetics of the two drugs to optimize synergistic anti-cancer effects. Similarly, our team constructed MMP-sensitive hydrogels to co-deliver DOX and aPD-L1 for postoperative treatment^202^. Because the positively charged chitosan delayed the release of the negatively charged aPD-L1, the sequential release pattern was caused by electrostatic interactions between payload and carrier. The consequent combination effect of ICB and ICD inhibited recurrence and metastasis in a 4T1 breast cancer model.

Glioblastoma multiforme (GBM), well-known as “the king of brain tumors”, still lacks mature immunotherapy regimens in clinical practice^223^^,^^224^. until the SDDSs were applied in postoperative immunotherapy, which brings good news to patients. Jiang et al. presented a hybrid oligopeptide hydrogel as a drug reservoir for co-delivery of tumor-homing immunomodulator (THINR) and CXCL10^214^. Mitoxantrone (MIT) and IDO1-targeting short interfering RNA (siIDO1) were loaded into THINR NPs. The release of CXCL10 from the gel was dominated by passive diffusion, whereas siIDO1 and MIT were prevented from diffusing due to physical encapsulation. Only when THINR is taken up by tumor cells can the drug be dissociated and released in the acidic endosomes. Initially released at a slow rate, CXCL10 continuously recruited circulating activated blood-borne T cells to the brain to attack residual tumor cells. MIT-induced ICD subsequently enhances T cell mediated immunity. siIDO1 overcame Treg-associated immunosuppression and further enhanced anti-tumor immunity by regulating tumor cell amino acid metabolism. *in vivo* results showed that SDDSs substantially improved the survival rate of the postoperative mouse glioma model compared to the no-gel co-release system. In conclusion, this sequential bionanomodulator-hydrogel ultrastructural drug delivery system holds promise as an alternative anti-tumor immune strategy for patients with resected brain tumors.

**Figure 6:**
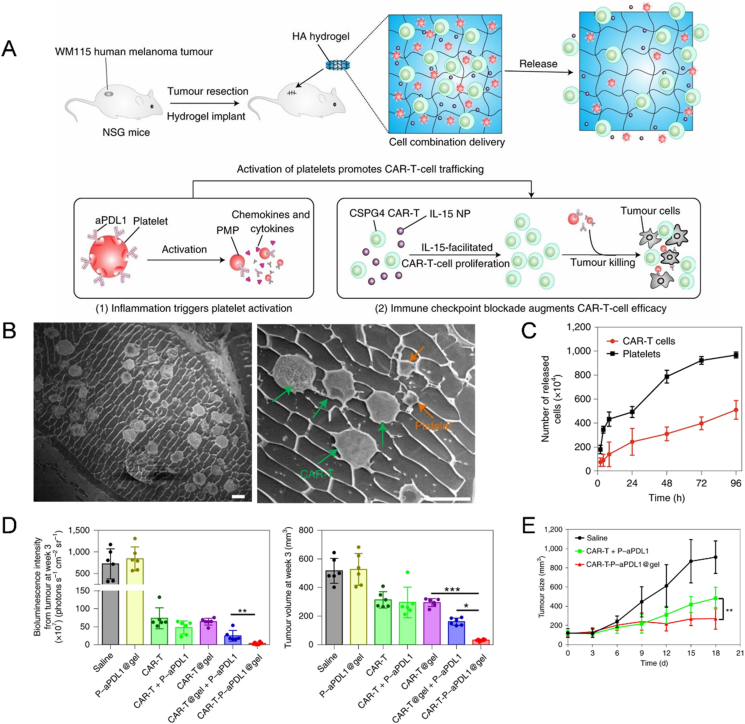
SDDS-based strategy for post-tumor resection therapy *via* immune enhancement. (A) Schematic illustration of a melanoma excision model and biodegradable HA hydrogel reservoir. Platelets activated during post-surgical wound healing release aPD-L1 in the form of PMP-aPD-L1. (B) Cryo-scanning electron microscopy imaging showed different sizes in CAR-T cells, platelets, and mesh. (C) Differential rates of CAR-T cell and platelet release *in vitro*. (D) CAR-T-P-aPD-L1@gel inhibited melanoma growth *in vivo*. (E) CAR-T-P-aPD-L1@gel facilitated abscopal anti-tumor effects. Reprinted with the permission from Ref. 209. Copyright © 2021 Springer Nature.

CAR-T cells are usually engineered for adoptive cell transfer by recognizing specific tumor antigens and subsequently killing cancer cells beyond the limitation of MHC restriction^225^. However, to present, it is still a tough mission to efficiently apply CAR-T cells in the treatment of solid tumors, where the immunosuppressive TME always leads to T cell rejection and depletion^226^. To address this defect, hu et al. developed a biodegradable HA hydrogel reservoir encapsulated with CAR-T cells targeting human chondroitin sulfate proteoglycan 4 (CSPG4), human platelet cells coupled to aPD-L1, and cytokine IL-15 NPs (Fig. 6A)^209^. The difference in release kinetics was caused by the fact that platelet cells were comparable to the mesh size of the gel, while CAR-T cells were larger than the mesh size (Fig. 6B and C). From an immunotherapeutic perspective, platelet cells were released from the gel and subsequently activated by the postoperative inflammatory milieu, which then secreted cytokines to recruit immune cells and amplify the anti-tumor effect. IL-15 NPs were designed to maintain the survival and proliferative capacity of CAR-T cells. This “sequential and sustained-release cell depot” delivery strategy represented a promising therapeutic approach for post-operative melanoma recurrence inhibition with abscopal effects (Fig. 6D and E).

#### 4.1.2 Reprogramming the soil to improve immunotherapy by immune normalization

TAMs are the most prevalent immune cells in the TME. Activated macrophages typically adopt one of two phenotypes: pro-inflammatory M1-like or anti-inflammatory, pro-repair M2-like^227^. M1 macrophages are crucial for antigen presentation and are capable of killing tumor cells and defending against pathogens. In contrast, M2 macrophages act as “espionage” among the immune cells, promoting tumor growth, invasion, and metastasis^228^^,^^229^. Since macrophages are plastic (M2-like macrophages in TAMs can be re-educated to the M1 phenotype), targeting TAMs represents a viable technique for reversing the immunosuppressive TME^230^. However, crafty cancer cells upregulate CD47 (a “don’t eat me” signal) on their surface, which interacts with SIRP*α* on M1-like macrophages and trigger tumor cells to evade recognition by macrophages^231^^,^^232^. Therefore, CD47 antibody (aCD47) can activate phagocytes, including M1-like macrophages, DCs, and neutrophils, improving their phagocytic capacity against cancer cells^233^, ^234^, ^235^, and then more effector T cells are activated to enhance the anti-tumor efficacy^236^.

**Figure 7:**
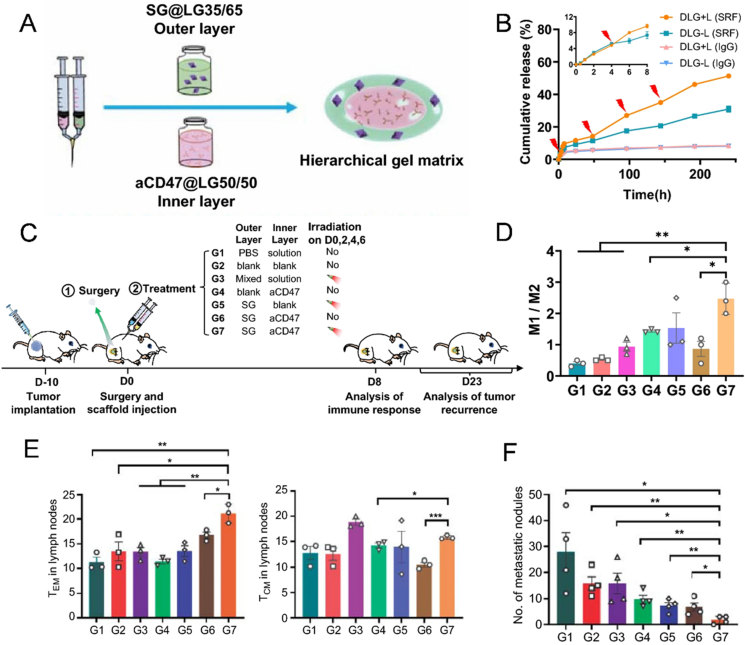
SDDS-based strategy for post-tumor resection therapy *via* immune normalization. (A) Preparation of hierarchical DLG matrix by a tailor-made dual syringe kit. (B) The release of SRF was accelerated by NIR irradiation. (C) Schematic diagram of the animal experimental design. (D) The ratio of M1/M2 in different treatment groups. (E) DLG matrix-based temporally programmed SDDSs facilitated the establishment of long-term anti-tumor immune memory. (F) Quantification of pulmonary metastatic nodules in mice of different treatment groups. Reprinted with the permission from Ref. 213. Copyright © 2021 Springer Nature.

Our team constructed a sequentially delivered bilayer structured lipid gel (DLG) to combine CD47 blockade and repolarization of TAMs^213^. The shell and core layers consisted of soybean glycerol dioleate (GDO) and phosphatidylcholine (SPC) with different mass ratios, which caused variable phase transition point temperatures. The SPC/GDO binary lipids system was biocompatible and allowed the drug release profile to be changed simply by adjusting the mass ratio (Fig. 7A)^237^. The shell layer was coated with graphene oxide NPs adsorbed on sorafenib, which generates heat under artificially controlled NIR irradiation to induce gel phase transitions for photo-controlled drug release (Fig. 7B). Sorafenib (SRF) was a small molecule kinase inhibitor that repolarized TAMs and promoted immunogenic TME^238^. The higher temperature of the core layer phase transition prolonged the sluggish release of aCD47 and inhibited the CD47/SIRPα axis, resulting in long-lasting anti-cancer effects. In a postoperative 4T1 model with poor immunogenicity and a high tendency for metastasis, the layered structure of DLG was shown to successfully raise the proportion of M1/M2 population and reduce suppressive TME (Fig. 7C and D). Remarkably, this local treatment also produced a systemic anti-tumor immunological memory, which prevented lung metastasis (Fig. 7E and F). In summary, the gel carrier that serves as an local drug delivery platform can fulfill time-series drug delivery and improve TME.

To reverse inhibitory TME by chemo-immunotherapy, our team designed a hybrid local depot to efficiently encapsulate cyclopamine (Cyc) and aCD47^204^. Cyc was distinguished from conventional chemotherapeutic agents by its ability not only to prevent tumorigenesis by specifically blocking aberrant activation of the hedgehog (Hh) signaling pathway in tumors, but also to inhibit ECM reconstruction at postoperative sites by interfering with the expression of ECM-associated proteins^239^^,^^240^. To improve the efficiency of Cyc uptake by tumor cells, it was encapsulated into cationic peptide R6-modified liposomes and subsequently co-loaded with aCD47 into a redox-sensitive hydrogel formed by cross-linking chitosan and pullulan. Due to the electrostatic repulsion between positively charged chitosan and positively charged liposomes, the release of Cyc-Lip was accelerated. When negatively charged aCD47 interacted with chitosan, it delayed release. In a postoperative mouse model with 4T1 tumors, this technique significantly outperformed local injections of free drugs in preventing recurrence, resisting re-challenge, and reducing metastasis. Thus, the SDDS-based combination strategy improved the efficacy of immunotherapy with CD47/SIRPα blockade. In another work, Chen et al. developed a sprayable bioresponsive fibrin gel for postoperative cancer treatment^215^. Biocompatible CaCO_3_ NPs served as both a proton scavenger to modulate the acidity of TME and a protective carrier for aCD47. The elevated pH led to repolarization of M2-like TAMs, and the subsequent release of aCD47 synergistically promoted anti-tumor immune responses. This straightforward administration encouraged a potential clinical application after tumor excision.

Another important type of immunosuppressive cell in TME is MDSCs that can be triggered by TLRs^241^. Through their ligands, TLRs can also boost the antigen-presenting function of DCs and the killing capacity of T lymphocytes. Phuengkham et al.^195^ designed an local postoperative scaffold formed by cross-linked collagen-HA hydrogels wrapped with DOX and R848-containing immune nanotransformers (iNCVs). DOX was slowly released due to hydrogen bonding and hydrophobic interactions between DOX and the polymer matrix. However, under the acidic circumstances of the tumor bed, the protonation of DOX’s amino group resulted in a weakening of the hydrogen bonds, hence accelerating its release rate. Nonetheless, due to the reduced particle size of DOX, its release rate was still somewhat slower than that of iNCVs. The early release of the immune adjuvant R848 induced the activation of APCs and the generation of antigen-specific T cells, thereby repolarizing MDSCs and M2-like TAMs towards an APCs phenotype. Slow-release DOX induced ICD and formed tumors into local vaccines. This combination strategy reprogrammed TME to an immunogenic phenotype, which subsequently enhanced anti-tumor immunity with ICB. Excellent anti-relapse and anti-metastatic effects were shown in postoperative models of 4T1 breast cancer and TC1 cervical cancer. This study demonstrated that polarization of immunosuppressive cells in TME into tumor suppressor cells was clearly superior to depletion.

### 4.2 Local drug delivery for intratumoral/peritumoral administration

Not all cancer patients are clinically suitable for surgical treatment due to various conditions, including: (1) Extensive metastasis where surgery is no longer effective. (2) Surgical removal of the tumor in the affected area is challenging. (3) Infiltrative cancer cells growing in multiple directions with unclear boundaries, making surgical removal impossible. (4) Coexistence of serious illnesses or health issues that render surgical treatment unfeasible. (5) Cancers that metastasize easily, such as undifferentiated small cell carcinoma of the lung, are generally not advocated for surgical treatment. Consequently, intratumoral/peritumoral administration is necessary for these patients. Moreover, the pivotal advantage of this local delivery modality is its significant reduction of toxic side effects commonly observed with systemic immunotherapy, rendering it safer and more effective^242^^,^^243^.

#### 4.2.1 Local injectable gel scaffolds

In recent years injectable gels have gained wide popularity in the field of tumor treatment due to the advantages of easy handling, minimally invasive nature and customizable release. Our team has proposed a “programmable all-in-one” injectable lipid gel to co-deliver the photosensitizer IR820 and an immune checkpoint inhibitor^237^. Initially, the lipid gel induced a transformation from “cold” tumors to “hot” tumors by recruiting immune cells through mild photothermal therapy (MPTT). Subsequently, the gel system achieved flexible control over the release of aPD-L1 by leveraging temperature-responsive phase changes. This strategy effectively weakened tumor immunosuppression, improved the TME, and promoted immune normalization.

Cancer cells generate ROS through multiple mechanisms, resulting in significantly elevated ROS levels in TME, which promotes tumorigenesis and progression. Thus, targeting ROS regulation for cancer therapy holds great promise^244^. Gu’s team created a ROS-responsive hydrogel for combination immunotherapy^194^. The injectable local hydrogel was prepared by cross-linking polyvinyl alcohol (PVA) and *N*^1^-(4-boronobenzyl)-*N*^3^-(4-boronophenyl)-*N*^1^,*N*^1^,*N*^3^,*N*^3^-tetramethylpropane-1,3-diaminium (TSPBA, a ROS-sensitive linker), which encapsulated gemcitabine (GEM) and aPD-L1. The essence of this platform was that the scaffold material itself acted as a ROS depleting agent, allowing for repolarization of TAMs. GEM was released considerably faster than aPD-L1 due to its lower molecular weight and reduced MDSCs. The synergy of GEM with the ROS-responsive gel promotes an immunogenic tumor phenotype and increases the number of TILs. Subsequently, later-released aPD-L1 amplified the effect of T cell response. Immune-mediated tumor regression was successfully induced in mouse models of B16F10 melanoma and 4T1 mammary tumors (low-immunogenic “cold” tumors that respond poorly to ICB). In conclusion, this sequential strategy better supported the natural course of immunity, overcame redundant immunosuppressive mechanisms, and was expected to be used in the future to treat metastatic tumors and inhibit tumor recurrence.

**Figure 8:**
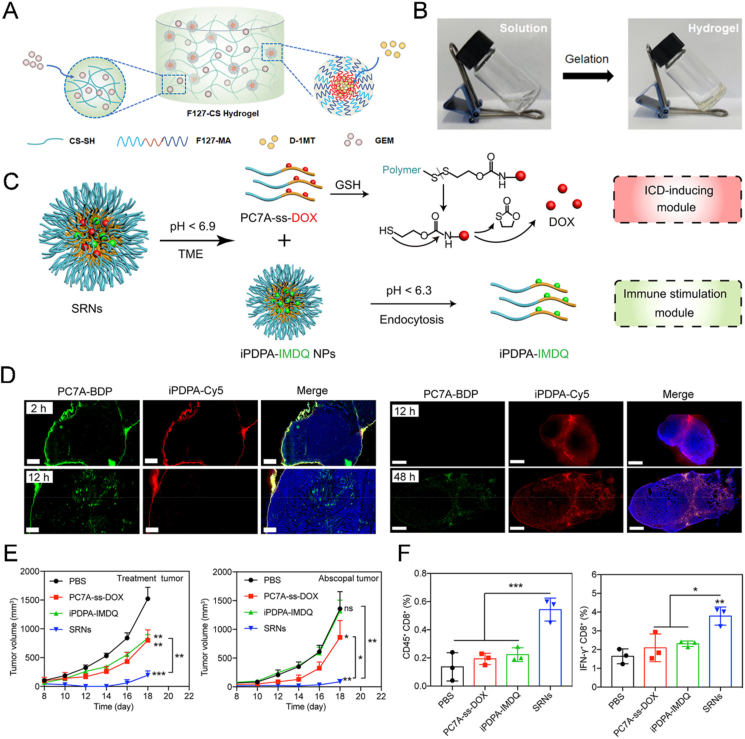
SDDS-based strategies for intratumoral/peritumoral administrations. (A) Schematic representation of F127 micelle-incorporated CS hydrogel with loaded D-1MT and GEM. (B) Photographs of the sol–gel transition of F127 micelles and CS-SH mixed solution. Reprinted with the permission from Ref. 196. Copyright © 2021 American Chemical Society. (C) Schematic representation of sequential pH/redox-responsive SRNs and drug release mechanism of PC7A-ss-DOX; PC7A-ss-DOX and iPDPA-IMDQ were considered as ICD-inducing and immunostimulatory modules, respectively. (D) Tumor (left) and lymph node (right) at 2, 12, and 48 h after peritumoral injection of PC7A-BDP/iPDPA-Cy5 hybrid NPs. (E) Growth curves of orthotopic tumors and abscopal tumors after different treatments. (F) The infiltration of CD8^+^ T cells was significantly enhanced in tumor tissues, implying strong activation of SLNs. Reprinted with the permission from Ref. 197. Copyright © 2021 American Chemical Society.

Indoleamine-2,3-dioxygenase (IDO) is an immunosuppressive enzyme that is commonly overexpressed in cancer cells and has been associated to tumor aggressiveness and unfavorable patient outcomes. Activation of the IDO pathway induces T cell deactivation or depletion through accumulation of the metabolite kynurenine, followed by immune escape from the tumor^245^^,^^246^. Thus, IDO is a promising target for immunotherapy^247^. Yu et al.^205^ established a ROS-degradable injectable peptide gel library for the sequential sustained release of aPD-1 and D-1MT (an IDO inhibitor). The gel formulation comprised a biocompatible triblock copolymer with a central PEG block flanked by ROS-responsive peptide blocks containing methionine and D-1MT. Typically, small molecules are released more rapidly than protein macromolecules, but here, D1-MT was attached to the gel skeleton by proteinase K-cleavable covalent bonds, resulting in the faster release of aPD-L1 than D1-MT. The drug-loaded hydrogel facilitates immune cell infiltration and potentiates anti-tumor effects when compared to the control group receiving an equivalent dose of free drug. Physical encapsulation of nanocarriers using gels is a typical method in addition to generating cleavable covalent bonds between the drug and the hydrogel substance^86^^,^^248^. Qin et al.^196^ constructed an injectable hydrogel doped with micelles that sequentially released GEM and D1-MT at the tumor site to achieve localized chemo-immunotherapy. D1-MT was encapsulated in a micelle core composed of amphiphilic methacrylated Pluronic F127 to address the poor water solubility (Fig. 8A). The micelles were subsequently reacted with thiolated chondroitin sulfate *via* a click reaction to form a hybrid hydrogel. Upon injection into the body, the hybrid hydrogel gelled and facilitated the delayed release of D-1MT (Fig. 8B). The hydrophilic GEM was dispersed in the hydrogel network, leading to rapid and early release. Ultimately, the hybrid hydrogel alleviated immunosuppressive TME and inhibited lung metastasis.

Temperature-sensitive hydrogels which have an appropriate gelation temperature at or below 37 °C are widely used for prolonged drug release^249^. However, once two or more therapeutics are loaded, how to make the drugs release in a pre-programmed manner is still a big challenge^250^, ^251^, ^252^. Li et al.^212^ integrated thermosensitive hydrogels and ROS-responsive nanogels into a TME-regulated peptide composite that allowed sequential delivery of LY3200882 (LY) and regorafenib (REG). LY was a selective TGF-*β* inhibitor that was encapsulated in a nanogel with a thioketal (TK) cross-linker (NG/LY). The nanogel was further mixed with REG and uniformly disseminated in a thermosensitive hydrogel consisting of methoxy poly(ethylene glycol)-*block*-poly(l-alanine) (mPEG*-b-*PAla) to form (Gel/(REG + NG/LY)). The released REG first inhibited tumor growth and promoted ROS formation in TME, and subsequently, ROS triggered the release of LY from (NG/LY). LY attenuated the enhanced metastatic capacity of tumor cells triggered by REG. In a mouse colorectal cancer model, (Gel/(REG + NG/LY)) showed superior inhibition of tumor growth compared to simultaneous administration of the individual components. The composite gel reduced the frequency of M2-like TAMs and MDSCs in TME, while increasing CTL level and effectively reversing immunosuppressive TME. Notably, LY blocked the TGF-*β* pathway, which may have prevented the EMT of tumor cells. The results of this study provided an effective strategy for intensive drug combination therapy, which holds potential to improve the prognosis of patients with advanced cancer.

In another study, Wu et al.^208^ prepared a temperature-sensitive hydrogel for loading IL-15 and cisplatin (CDDP). This injectable hydrogel was consisted of poly (ethylene glycol) monomethyl ether-poly(*γ*-ethyl-l-glutamate) (mPEG*-b-*PELG) diblock copolymer. *in vitro* release studies, gels exhibited biphasic and sustained release profiles, which correlated with the drug release kinetics. The authors attributed this observation to the enhanced water solubility of the small molecule CDDP in PBS and the slower diffusion rate of the protein. Additionally, a weak interaction may exist between IL-15 and the polypeptide chain. In a mouse model of melanoma, this SDDS-based local hydrogel exhibited synergistic therapeutic benefits and attained satisfactory survival rates.

#### 4.2.2 Stimuli-responsive sequential delivery nanocarriers

Sentinel lymph nodes (SLNs) are part of the TdLNs and represent the first lymph nodes that cancer cells must encounter during lymph node metastasis from the primary tumor. In comparison to intravenous administration, intratumoral delivery leads to higher drug concentrations in TdLNs^253^. Due to the limited activation of immune cells within the lymph nodes, Wang et al.^197^ deemed ICD-triggered TME remodeling to be an unsatisfactory treatment. Concurrent delivery of antigens and pattern recognition receptor agonists to SLNs in parallel the induction of ICD could potentially evoke a potent anti-tumor response both local and distally. To achieve this goal, researchers constructed sequential pH/redox-responsive NPs (SRNs) for TME and DC endosomal sequential responses^197^. The SRNs consisted of two modules: PEG*-b-*P (C7A-r-ss-DOX) and PEG*-b-*P (iPDA-r-IMDQ) polymer-drug conjugates. TME-targeted module I induced ICD, while SLN-targeted module II was responsible for immune activation. Module I can be rapidly dissociated in TME due to the dual responsiveness of pH 6.9 and reducibility. Module II was designed to dissociate at a much lower pH 6.3 (Fig. 8C). The DOX released first induced a full ICD at the tumor site and then reversed the inhibitory TME by generating a broad spectrum of tumor antigens. Subsequently, module II flowed into the SLNs, along with the antigens to be taken up by DCs. Imidazoquinolines (IMDQs, TLR7/8 agonists) released in the endosome activated DCs and facilitated antigen presentation. Immunofluorescence analysis showed robust tumor deep penetration and lymph node drainage by SRNs (Fig. 8D). This spatiotemporally programmed delivery strategy reduced systemic toxicities and enhanced the initial three phases in the cancer–immunity cycle. Eventually, robust immune activation was demonstrated in melanoma and colorectal cancer models (Fig. 8E and F). Altogether, this ultra-pH-sensitive (UPS) nanotechnology platform provided a new paradigm for the design of SDDSs.

A shell–core structure of SDDSs is another practicable approach to achieving sequential release. Chen et al. developed a ROS-sensitive protein complex for combination immunotherapy^199^. The poorly immunogenic albumin was chosen as the delivery material to prevent excessive cross-linking, which would lead to inactivation of the antibody. The complex used a ROS-responsive linker and aCD47/aPD-1 in the outer/inner layer. Initially, aCD47 sufficiently activated the innate immune system to recognize tumors and launch T cell attacks. Subsequently, aPD-1 reduced T cell deactivation and increased T cells population. The carrier itself also depleted ROS to down-regulate immunosuppressive pathways in TME, thereby reducing TAMs and Treg. In another study, Jia et al.^198^ constructed a pH-responsive pickering nanoemulsion (PNE) for sequential co-delivery of DOX and ICI through a shell–core structure. The formulation’s key feature was the use of multi-sensitive nanogels (SNG) that respond to pH changes, hydrophilic-hydrophobic shifts, and glutathione (GSH), acting as stabilizers at the oil-water interface. The oil-soluble small molecule HY (PD-1/PD-L1 inhibitor) was encapsulated into the inner oil phase of the PNE, and DOX was loaded as a shell layer onto the SNG to form D/HY@PNE. Upon intratumoral injection of D/HY@PNE, the hydrophilic-hydrophobic transition of the SNG led to the release of HY and DOX-loaded SNG. SNG’s tiny size and hydrophobicity made it more easily absorbed by tumor cells. The release of DOX from SNG triggered ICD due to high levels of GSH within tumor cells. Subsequently, HY enhanced T cell immunity by blocking the PD-1/PD-1 axis. Overall, this strategy provided a versatile co-delivery platform to achieve spatiotemporal delivery of different drugs.

## 5 Conclusion and prospects

Despite tremendous developments in the last decade, tumor immunotherapy still suffers from low response rates to monotherapy and irAEs. Our object is to restart and maintain the cancer–immunity cycle by combining immunotherapies, which can then exert efficient anti-tumor activity while decreasing systemic toxicities *via* designing a spatiotemporal delivery strategy of SDDSs. Here, we first classify the targets of cancer–immunity cycle for SDDSs into immune enhancement and immune normalization, and then enumerate and discuss the application of SDDSs in both systemic and local administration for both types of directions. Although these strategies have demonstrated promising efficacy in preclinical models, there are still several factors that need to be taken into consideration.

To begin, the dosage of drug in reservoirs for local administration and in chemo-immunotherapy carriers for systemic administration should be carefully evaluated in addition to their inherent stability and biocompatibility. It is of the utmost importance to determine the optimal ratio and dosage of drugs for co-delivery, given that accurate dosing can augment therapeutic efficacy and improper dosing may lead to ineffectiveness or toxic side effects. Furthermore, the extent to which differences in drug release kinetics will affect the effectiveness of this therapy also needs to be examined. Secondly, for photo-immunotherapy, the limited depth of laser exposure is a typical issue that requires immediate attention, in addition to the potential toxicity risk of photosensitizers and photothermal agents, which needs to be rationally evaluated. In photodynamic-immunotherapy, it is still a big challenge to quantitatively determine the excitation level of ROS *in vivo* and assess its impact on the immune activity. In photothermal–immunotherapy, the effects of immunomodulated thermal dosage (a function of temperature and exposure time) are currently largely understudied. To optimize therapeutic efficiency, investigators need to evaluate additional carrier platform-based temperature effects *in vivo*. Lastly, sequential gene-immunotherapies are still in their infancy and have received little attention. Future research should focus on evaluating immune-related side effects caused by off-target effects of siRNA within the complex *in vivo* environment.

In addition to concerns against various tumor combination immunotherapies, biomaterial-based SDDS still face many challenges in clinical translation. First, tumor development is accompanied by a wide range of physiological changes^254^, including abnormal cell proliferation, vascular and lymphatic vessel abnormalities, genomic instability, and epigenetic alterations. Different tumors have different characteristics of changes, and this heterogeneity poses a great obstacle to the design of SDDS. Therefore, future SDDS design must take into account each tumor and its unique physiology and ecological niche. For example, comprehensive analysis of tumors and their microenvironments by single-cell RNA sequencing provides insight into patient-specific somatic mutations to hypothesize defects in the cancer–immunity cycle^255^. SDDS based on this design can restore the cancer–immune cycle and stimulate a robust anti-tumor immune response. Second, the impact of prior cancer treatment on SDDS is another major challenge. For instance, most patients undergo surgery and postoperative adjuvant therapy, and surgery may have altered their physiologic status and immune function. Therefore, when validating SDDS for tumor combination immunotherapy, the responsiveness of immunotherapy needs to be pre-tested at the cellular level to avoid ineffective treatment. In addition, clinically validated sequential therapeutic regimens may further improve clinical translation and therapeutic efficacy if they are combined with SDDS strategies.

On the other hand, the safety of SDDS carrier materials is also a key issue. Non-degradable biomaterials may trigger toxic side effects and rejection reactions, so ideal carrier materials should have biodegradability, low immunogenicity and good biocompatibility. Currently, PLGA-based scaffold vaccines have entered phase I clinical trials for stage IV melanoma and are licensed for commercialization by Novartis, and they can be used as a safe therapeutic platform for a variety of tumors. Other marketed microscopic carrier platforms, such as lipid nanoparticles, albumin nanoparticles and degradable PLGA microspheres, also inform SDDS design. Meanwhile, natural materials such as extracellular vesicles, hyaluronic acid, chitosan and collagen are more suitable as SDDS carriers due to their greater safety. The study of immune response, metabolism and clearance mechanisms of biomaterials is particularly important in clinical testing, so these safety issues must be fully considered in the early stages of SDDS design to ensure efficacy and minimize side effects. Finally, the issue of mass production of carrier materials should not be overlooked. It is best to select materials that have been approved by the FDA, are easy to produce in high throughput, and have low batch variation. This need for standardization has been demonstrated in the rapid development, validation, production and application of COVID-19 mRNA vaccines.

Overall, SDDSs overcome the shortcomings of other therapeutic tools in terms of temporal and spatial control of the drug and provide tremendous success and new insights into multiple aspects of tumor immunotherapy. We hope that this review will stimulate further interest in tumor immunotherapy and foster interdisciplinary collaborations between basic biology and engineering to develop more novel and valuable strategies for SDDS-mediated immunotherapy.

## Author contributions

Zhenyu Xu: Writing – original draft, Methodology, Investigation. Siyan Liu: Writing – original draft, Investigation. Yanan Li: Writing – original draft. Yanping Wu: Writing – original draft. Jiasheng Tu: Writing – review & editing, Supervision, Resources, Conceptualization. Qian Chen: Writing – review & editing, Supervision, Resources, Conceptualization. Chunmeng Sun: Writing – review & editing, Supervision, Resources, Conceptualization.

## Conflicts of interest

The authors have no conflicts of interest to declare.
